# Dissecting Epigenetic Silencing Complexity in the Mouse Lung Cancer Suppressor Gene *Cadm1*

**DOI:** 10.1371/journal.pone.0038531

**Published:** 2012-06-06

**Authors:** Stella Marie Reamon-Buettner, Juergen Borlak

**Affiliations:** Toxicology and Environmental Hygiene, Fraunhofer Institute for Toxicology and Experimental Medicine, Hannover, Germany; University of Navarra, Spain

## Abstract

Disease-oriented functional analysis of epigenetic factors and their regulatory mechanisms in aberrant silencing is a prerequisite for better diagnostics and therapy. Yet, the precise mechanisms are still unclear and complex, involving the interplay of several effectors including nucleosome positioning, DNA methylation, histone variants and histone modifications. We investigated the epigenetic silencing complexity in the tumor suppressor gene *Cadm1* in mouse lung cancer progenitor cell lines, exhibiting promoter hypermethylation associated with transcriptional repression, but mostly unresponsive to demethylating drug treatments. After predicting nucleosome positions and transcription factor binding sites along the *Cadm1* promoter, we carried out single-molecule mapping with DNA methyltransferase *M.Sss*I, which revealed in silent promoters high nucleosome occupancy and occlusion of transcription factor binding sites. Furthermore, *M.Sss*I maps of promoters varied within and among the different lung cancer cell lines. Chromatin analysis with micrococcal nuclease also indicated variations in nucleosome positioning to have implications in the binding of transcription factors near nucleosome borders. Chromatin immunoprecipitation showed that histone variants (H2A.Z and H3.3), and opposing histone modification marks (H3K4me3 and H3K27me3) all colocalized in the same nucleosome positions that is reminiscent of epigenetic plasticity in embryonic stem cells. Altogether, epigenetic silencing complexity in the promoter region of *Cadm1* is not only defined by DNA hypermethylation, but high nucleosome occupancy, altered nucleosome positioning, and ‘bivalent’ histone modifications, also likely contributed in the transcriptional repression of this gene in the lung cancer cells. Our results will help define therapeutic intervention strategies using epigenetic drugs in lung cancer.

## Introduction

Lung cancer remains a leading cause of death, but the molecular mechanisms of disease are largely unknown. Many studies now show that genetic and epigenetic alterations as culprits [1]. Epigenetic events are heritable changes in gene expression without alterations in primary DNA sequence. They are important in normal development and differentiation, but when misdirected lead to diseases, notably cancer [2]. Nonetheless, many of the processes resulting in gene silencing can be reversed with epigenetic drugs, offering a hope for treatment and therapy [3]. The epigenetic landscape of silencing is, however, complex involving the interplay of major effectors including nucleosome positioning, DNA methylation, histone variants, histone modifications and non-coding RNAs [4]. How these effectors interact to each other to affect gene expression and cause disease remains unclear.

The DNA is packaged into a complex nucleoprotein structure in the nucleus of a cell called chromatin, and the basic repeating unit of chromatin is known as nucleosome, the structure and function of which are still being elucidated [5]. Each nucleosome consists of an octameric histone core (two copies each of H2A, H2B, H3, and H4), around which approximately 147 bp of DNA are wrapped in 1.65 superhelical turns. Nucleosome positioning plays a crucial role in chromatin higher order folding and in gene regulation [6]–[8]. Nucleosomes can affect transcription by modulating the accessibility of DNA to regulatory proteins and transcriptional machinery, leading to gene activation or repression. Nucleosome positioning can, in turn, be affected by several factors, including DNA sequence preferences, DNA methylation, histone variants, and histone posttranslational modifications [6]. Moreover, nucleosome positioning differs from nucleosome occupancy, which does not account nucleosome starts provided that a given base pair is inside a nucleosome [7].

Modification by DNA methylation occurs by the covalent addition of a methyl group to position 5 of the cytosine ring, creating 5-methylcytosine. DNA methylation is a well-known epigenetic silencing mechanism and is associated in various biological processes and diseases (reviews, [4], [9]). Tet (ten eleven translocation) proteins can convert 5-methylcytosine (5mC) into 5-hydroxymethylcytosine (5hmC) [10], [11], and recently also into 5-formylcytosine (5fC) and 5-carboxylcytosine (5caC) [12]. DNA methylation may inhibit gene expression by preventing transcriptional activators from binding the DNA target or by recruitment of methyl-CpG-binding domain (MBD) proteins, which in turn recruit histone-modifying and chromatin-remodelling complexes to methylated sites [4]. CpG methylation may also contribute to the repression of gene by inducing a more compact and rigid nucleosome conformation [13].

The mammalian DNA methylation machinery is mediated by the DNA methyltransferases (DNMTs), which establish and maintain DNA methylation patterns. DNMT1 is required in maintaining DNA methylation patterns, while *de novo* methyltransferases DNMT3A and DNMT3B target new unmethylated DNA sites (for review, [14]). Nucleosomes can influence DNA methylation, but so far studies show contrasting results. Either DNA methyltransferases preferentially target nucleosome-bound DNA [15], or nucleosomes render protection against methylation [16]. Furthermore, nucleosomes containing methylated DNA stabilize de novo DNA methyltransferases 3A/3B (DNMT3A/3B) allowing little free DNMT3A/3B to exist in the nucleus [17]. Stabilization of DNMT3A/3B on nucleosomes in methylated regions further promotes propagation of DNA methylation and thus ensures faithful epigenetic inheritance. CpG methylation can also have a distinct influence on protein binding when it is present within a nucleosomal background [18].

Nucleosomal histones can be exchanged with histone variants, and their incorporation can influence nucleosome positioning, and thus gene activity (reviewed in [19]). The synthesis of canonical histones is coupled to DNA replication in S phase, while histone variants are synthesized throughout the cell cycle. Furthermore, in contrast to canonical histones whose function is primarily in genome packaging and gene regulation, non-canonical histones have crucial roles in a range of processes, including chromosome segregation, transcriptional regulation, and DNA repair. Among these histone variants is the H2A variant H2A.Z, which is highly conserved during eukaryotic evolution [20], [21]. Histone variant H2A.Z differs significantly from H2A by several amino acids and preferentially localizes to gene promoters in mammalian cells, where it is spread over several nucleosomes upstream and downstream of transcription start site [22]. Another well-conserved histone variant is H3.3, a variant that differs from canonical H3 by few amino acid substitutions, and found to be enriched throughout the gene body of transcribed genes, promoter regions in active and inactive genes, and at regulatory elements. This variant also accumulates at silent loci in pericentric chromatin and telomeres [23].

Besides replacement of histone variants, amino acid residues in the N-terminal tails of histones (canonical as well as variants), can be modified by various covalent post-translational modifications (PTMs) and form the basis for the epigenetic regulation of chromatin structure and gene function [24]. More important, there is crosstalk between histone modifications with other epigenetic regulators to reinforce or reverse functions of modifications. Such PTMs, which include among others acetylation, methylation, and phosphorylation, play a direct role in affecting chromatin structure, or they may represent marks or signals to be recognized by readers of histone modifications, to specify a loose or compact chromatin [25]. Disruptions of histone modifications in normal regulatory processes have been found in diseases, including cancer development and progression [26].

*CADM1* (cell adhesion molecule 1) is a tumor suppressor gene identified in non-small cell lung cancer (NSCLC), but also implicated in other human cancer diseases [27], [28]. We showed previously that *Cadm1* was repressed in mouse lung cancer progenitor cell lines, and gene expression highly correlated with promoter hypermethylation [29]. But after treatment with the demethylating agent 5-aza-2-deoxycytidine (5-aza-dC), most of the cell lines were not responsive suggesting the participation of additional epigenetic silencing events. This present study aimed to understand epigenetic landscapes leading to transcriptional repression of *Cadm1* in the same mouse lung cancer progenitor cells, and eventually to gain mechanistic insights into the epigenetic silencing of tumor suppressor genes, in general, and response to epigenetic drugs. Using bioinformatic tools, we predicted nucleosome positions and transcription factor binding sites along the *Cadm1* promoter. We carried out a rigorous single-molecule mapping of chromatin with the DNA methyltransferase, *M.Sss*I to determine nucleosome occupancy and occlusion of transcription factor binding sites. With a panel of primers to interrogate the middle, as well as left and right borders of predicted nucleosomes, we analyzed for differential nucleosome positioning in MNase-digested chromatin and ChIPed DNA with canonical histone H2A, histone variants H2A.Z and H3.3, as well as for histone modifications, H3K4me3 and H3K27me3. Altogether, the lung cancer cells displayed several epigenetic silencing events that will help to define therapeutic intervention strategies.

## Results

### *Cadm1* Promoter Hypermethylation Correlates with Transcriptional Repression

This and previous study [29], found that *Cadm1* promoter CpG hypermethylation correlated with transcriptional repression in lung cancer cell lines established from single, spontaneously transformed lung tumor cells of *c-Myc* and *c-Raf* double-transgenic mice. CpG methylation in individual clones was heterogeneous within and among the 10 different lung cancer cell lines. That previous study also demonstrated that methylation of CpGs in the core binding sites of transcription factors Sp1, Sp3, and Zf5 abrogated DNA-protein binding. Furthermore, treatment with the demethylating agent, 5-aza-2-deoxycytidine (5-aza-dC) restored *Cadm1* gene expression, but so far only in two cell lines, suggesting additional epigenetic silencing events. Indeed, comparing DNA methylation in untreated and in aza-treated lung cancer cell line A2C12 with corresponding re-expression of *Cadm1* showed that most of the 69 CpGs analyzed in the promoter region were still methylated in the aza-treated A2C12. Thus, treatment with 5-aza-dC alone was not able to reinstate gene expression in all the lung cancer cell lines or demethylate the promoter region of *Cadm1* and these observations led us to suspect for additional layers of epigenetic silencing in place. We further investigated the *Cadm1* promoter and thereby to gain insights into the extent of the epigenetic silencing complexity in the different lung cancer cell lines.

### Nucleosome Positioning Predictions and Annotations along the *Cadm1* Promoter Region

**Figure 1:**
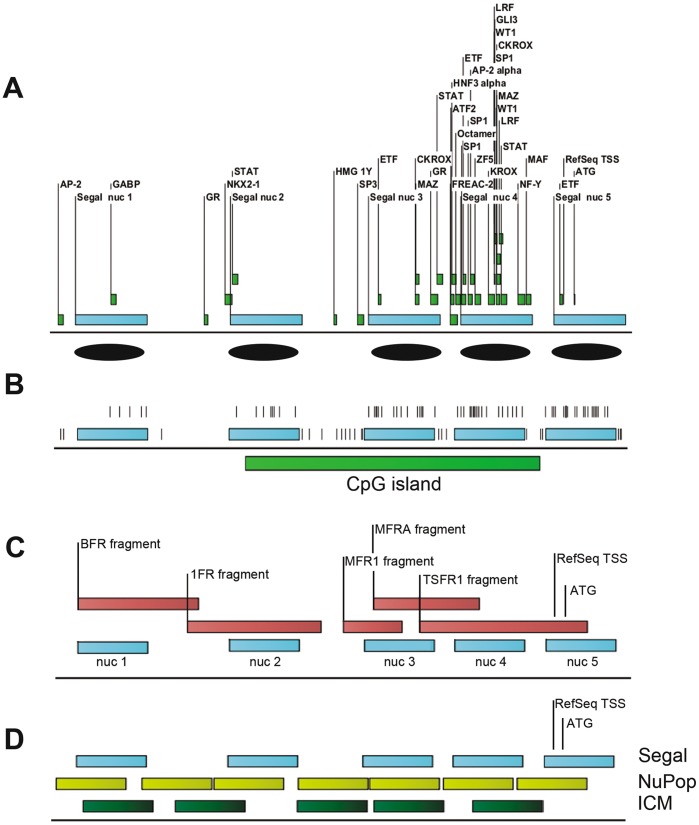
Genomic DNA sequence-based bioinformatic predictions of nucleosome positions and transcription factor binding sites along the promoter region of *Cadm1*. (**A**) Position of five analyzed nucleosomes (blue rectangles) and binding sites of transcription factors. Nucleosomes are arbitrarily numbered starting from the farthest (e.g. nuc 1) towards the RefSeq transcription start site (TSS) and the translation start site ATG, which are both located at nucleosome 5 (nuc 5). Nucleotide numbering with +1 corresponds to A of the ATG. (**B**) Predicted nucleosomes and location of CpGs (vertical stripes) and the CpG island along the *Cadm1* promoter. (**C**) Five fragments covering analyzed CpGs in bisulfite-treated genomic DNA and predicted nucleosomes. (**D**) Possible nucleosome positions from three different algorithms.

To determine whether CpG methylation could influence nucleosome occupancy leading to epigenetic silencing, as previously shown [30], we used bioinformatic tools to predict nucleosome positions, and to annotate the *Cadm1* promoter region. Using a nucleosome positioning prediction based on genomic DNA sequence (Segal, see Materials and Methods), we located at least five possible nucleosome positions approximately 1000 bp towards the transcription start site (TSS) and the translation start site (ATG). The RefSeq TSS (NM_001025600.1) is located at –21 of ATG. The predicted nucleosomes are designated arbitrarily relative to the ATG, as nuc 1 (−1011 to −865), nuc 2 (−697 to −551), nuc 3 (−417 to −271), nuc 4 (−230 to −84) and nuc 5 (−41 to +106). The binding sites of predicted sequence-specific transcription factors lie at the borders or within these nucleosomes, and highly concentrated at the nucleosomes most adjacent to the TSS (**Figure 1A**). Many CpGs are found inside nucleosomes, especially those within the CpG island in the promoter region of *Cadm1* (**Figure 1B**). The 1000-bp region is covered by five fragments of sizes 124–349 bp we used to analyze CpG methylation in bisulfite-treated genomic DNA, particularly in conjunction with DNA methyltransferase-based single-molecule chromatin (MAP-IT) assay (**Figure 1C**, **Table S1**). During the study, other nucleosome positioning algorithms became available (e.g. NuPOP, ICM, see Materials and Methods) and comparison showed overlap in predictions among the three methods used (**Figure 1D**). Nonetheless, the position of three nucleosomes (designated nuc 1, 3, 4) appeared to be more or less consistent.

### Single-molecule Chromatin Mapping of *Cadm1* Promoter Region with *M.SssI* Reveals High Nucleosome Occupancy in Lung Cancer Cells

We next conducted a single-molecule chromatin mapping of the *Cadm1* promoter region. We utilized a footprinting strategy that enables chromatin structure mapping at unmethylated CpG islands by treating isolated nuclei with DNA methyltransferases, notably the CpG-specific DNA methyltransferase (*M.Sss*I), followed by genomic bisulfite sequencing of individual progeny DNA molecules [31], [32]. This procedure termed as methylation-based single promoter analysis (M-SPA), is also described as methyltransferase accessibility protocol for individual templates (MAP-IT) [33]. Essentially, CpGs will be methylated by *M.Sss*I, a bacterial cytosine C5 methyltransferase, unless the CpGs are blocked by nucleosomes or DNA binding proteins. To this effect, nucleosome localization is defined as a region of about 147 bp that is inaccessible to *M.Sss*I.

The CpGs within the *Cadm1* promoter region in normal lung are essentially unmethylated and the gene is expressed. We treated the chromatin of normal lung with *M.Sss*I and analyzed protected (unmethylated) CpGs along the promoter region of *Cadm1*, especially those within predicted nucleosomes or transcription factor binding sites. We used bisulfite primers that amplify five fragments, three of which overlap, and cover 69 CpGs from −944 to +41, relative to the translation start site, ATG (see **Figure 1C** **, Table S1**). *M.Sss*I treatment of ‘naked’ genomic DNA served as control. Using the DNA methylation pattern in ‘naked’ genomic DNA and normal lung as reference, we analyzed chromatin from lung tumor, lung cancer cell lines, and a 5-aza-dC-treated lung cancer cell line with slight gene re-expression. To gain insights of different snapshots of the *Cadm1* promoter, we compared DNA methylation patterns of independent *M.Sss*I treatments of same cell lines.

#### Methylation efficiency of M.SssI along the Cadm1 promoter region in ‘naked’ genomic DNA controls

Methylation efficiency of *M.Sss*I was first determined in ‘naked’ mouse genomic DNA. The methylation efficiency in average 21 clones for each fragment ranged from 51–91% **(Figure S1)**. The highest efficiency was obtained in the two closest fragments around the TSS, i.e. MFRA and TSFR1 at 91%. Unmethylated CpGs in these fragments were more or less random. This differential methylation efficiency could be an indication that the promoter region in the vicinity of TSS was more sensitive to DNA methylation. In our previous analysis, methylation index at fragment TSFR1 that includes the TSS gave the clearest correlation to transcriptional repression. Overall methylation in five fragments, 106 clones and 1,478 CpGs was 76%. Using the same protocol, we also determined the methylation efficiency of ‘naked’ genomic DNA isolated from a lung cancer cell line (A2Cl2) which already contained prior CpG methylation. Overall methylation in five fragments, 31 clones and 416 CpGs was 98%, indicating robustness of the assay.

#### M.SssI chromatin map of normal lung

We analyzed the M.SssI map in chromatin isolated from nine pooled normal lungs. For fragment BFR (255 bp, 6 CpGs −944 to −837), most clones showed a stretch of unmethylated CpGs **(Figure S1)**, especially those which are located within a predicted nucleosome (nuc 1) **(Figure S2)**. For fragment 1FR (279 bp, 10 CpGs, −682 to −531), several clones also showed a stretch of unmethylated CpGs, many fall within a predicted nucleosome (nuc 2) **(Figure S3)**. These results are suggestive of nucleosome occupancy.

For the next three overlapping fragments around the TSS (MFR1, MFRA, TSFR1) and in the region where several transcription factor binding sites could be found (see **Figure 1A**), the patterns in most clones indicated absence of nucleosome occupancy, but rather suggestive of binding of transcription factors or other proteins necessary for regulation. Fragment MFR1 (124 bp, 14 CpGs, −456 to −341) is amplified by methylation-specific primers (with three CpGs in both forward and reverse primers). This fragment contains predicted binding sites for transcription factors, for example PPARg, ER, ETF, in which the CpGs within these binding sites were mostly unmethylated in several clones, to suggest their binding and a possible role in the transcriptional regulation of *Cadm1*. The predicted ETF site is inside a nucleosome (nuc 3), while the PPARg is at the border of nuc 3, and could be readily influenced by alterations concerning the nucleosome occupancy and sliding **(Figure S4).**

Fragment MFRA (222 bp, 27 CpGs, −396 to −180) is also amplified by methylation-specific primers (with 5 CpGs in forward primer, 6 CpGs in reverse primer). This fragment covers a predicted nucleosome (nuc 3), and partly that of another (nuc 4) **(Figure S5)**. It contains two binding sites for Sp1 which are located within or at the left border of nuc 4. The CpGs in the Sp1 binding sites at −224 and −211 were occupied in many clones having nucleosome-free pattern. This result supports further that Sp1 may indeed play a role in the regulation of *Cadm1*. There were 2 of 15 clones, however, which showed a long stretch of CpG protection inside a predicted nucleosome (nuc 3), indicating nucleosome formation in this part of the promoter of *Cadm1*.

**Figure 2:**
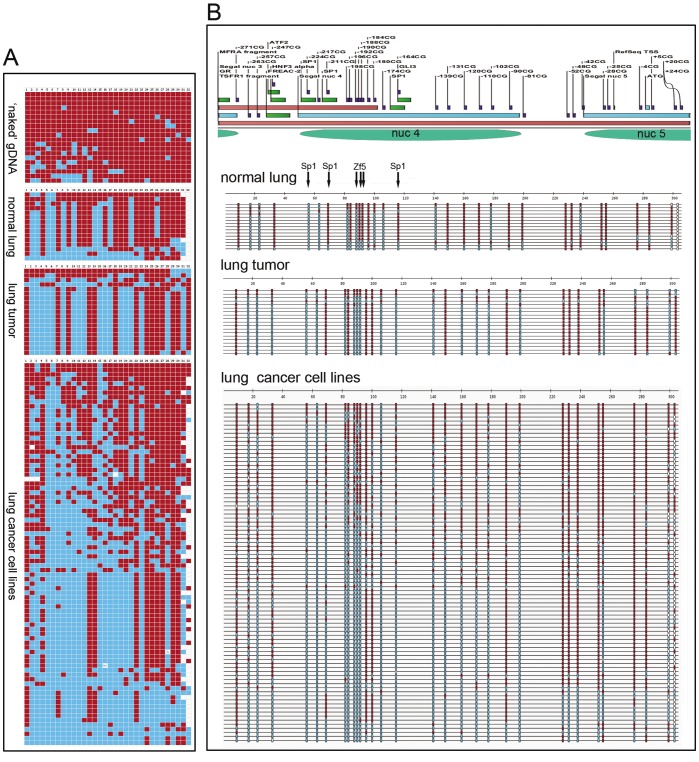
DNA methyltransferase-based single-molecule chromatin (MAP-IT) assay of *Cadm1* promoter region. (**A**) Methylation patterns in clones after treatment with CpG-specific DNA methyltransferase (*M.Sss*I) and scoring of 32 CpGs (−271 to +24 CpGs, TSFR1 fragment) in ‘naked’ mouse-tail genomic DNA, and chromatin from nine pooled normal lungs, three pooled solid lung tumors, and seven different lung cancer cell lines with little or no *Cadm1* gene expression. The patterns were obtained with BISMA where blue boxes representing unmethylated CpGs ( = protected) while red boxes, methylated CpGs. In lung tumors and lung cancer cell lines, CpG methylation could be endogenous and/or from the *M.Sss*I treatment. (**B**) Annotation of analyzed *Cadm1* promoter region (CpGs, putative binding sites of lung-specific transcription factors, predicted nucleosomes), and the corresponding sequence-context DNA methylation patterns shown in (**A**). A stretch of protected CpGs especially within the predicted nucleosome 4 was frequent in many of the 84 clones obtained in lung cancer cell lines.

Fragment TSFR1 (345 bp, 37 CpGs, −302 +41) is amplified by primers that contain three CpGs on the forward primer, and two CpGs in the reverse primer (**Figure 2**). Our previous results showed that the methylation index obtained from this fragment correlated highly with transcriptional repression as compared with the other fragments analyzed in the *Cadm1* promoter region. It contains binding sites for Sp1, Zf5, and other predicted transcription factors. Included also are the RefSeq transcription start site (TSS), the translation start site, ATG as well as at least two predicted nucleosomes (nuc 4 and nuc 5). There was no long stretch of unmethylated CpGs, except for 3 of 15 clones in which most of the protected CpGs fall within a predicted nucleosome (nuc 4). In those clones without apparent occupancy of nuc 4, a predicted nucleosome (nuc 5) where the RefSeq TSS as well as the ATG sites are located, appeared to be not present as well, consistent of an open chromatin that is associated with transcription. Furthermore, the −224 CpG in the binding site of Sp1 and the −192 CpG in Zf5, were frequently unmethylated, to support their binding in the promoter region of *Cadm1.*

To summarize, the *M.Sss*I methylation map observed in clones of normal lung suggested the formation of the predicted five nucleosomes along the promoter region of *Cadm1*. The patterns found in the three fragments (MFR1, MFRA, and TSFR1) around the TSS, in which three nucleosomes (nuc 3, nuc 4, and nuc 5) are located, showed the absence of nucleosome occupancy in many clones. However, the binding sites of transcription factors such as Sp1 and Zf5 showed protection, suggesting their role in the transcriptional regulation of *Cadm1*. In contrast, for the two nucleosomes farther away from the TSS (nuc1 and nuc 2), no apparent nucleosome remodeling appeared to take place as most clones only exhibited long stretches of unmethylated CpGs.

#### M.SssI chromatin map of lung tumor

We analyzed the *M.Sss*I map of chromatin isolated from three pooled solid lung tumors of c*-Raf* transgenic mice. *Cadm1* was still expressed in these tumors (not shown). Owing to endogenous CpG methylation in the lung tumors, only results from unmethylated CpGs (i.e. protected) after *M.Sss*I treatment were interpreted. Nonetheless, patterns in clones suggested the formation of at least three nucleosomes (nuc1, nuc 2, nuc 3) **(Figures S1, S2, S3, S5)**. At fragment TSFR1, where nuc 4 and nuc 5 reside, there was no long stretch of unmethylated CpGs along the fragment to indicate nucleosome occupancy, but 18 individual CpG sites showed protection (**Figure 2**). Indeed, 13 of 19 clones exhibited the same pattern. Among the features of these common pattern include **almost** no methylation in two Sp1 binding sites (−224, −164 CpGs), as well as that of Zf5 (−192, −190, −188 CpGs). Treatment of chromatin derived from the lung tumor sample did not show the presence of nucleosomal occupancy in analyzed clones at fragment TSFR1. However, this result was based on pooled lung tumors that still expressed to some degree *Cadm1*. Furthermore, lung tumor is composed of many cell types including non-cancerous ones which might have contributed to the findings shown in **Figure 2**. Nonetheless, at the level of individual tumor cell lines, marked differences in the nucleosomal positioning were observed as discussed below.

#### M.SssI chromatin maps of different lung cancer cell lines

We carried out *M.Sss*I mapping on 10 lung cancer cell lines with varying degrees of *Cadm1* promoter hypermethylation and transcriptional repression, as well as a lung cancer cell line treated with 5-aza-dC. Similar to lung tumor, because of endogenous CpG methylation in the lung cancer cell lines, only patterns from unmethylated CpGs (i.e. protected) after *M.Sss*I treatment were considered. We analyzed maps of individual cell lines **(Figure S6, S7)** as well as collectively. As earlier mentioned, we also analyzed patterns in ‘naked’ genomic DNA from a lung cancer cell line (A2C12), which was endogenously methylated (see **Figure S1C**). Furthermore, to confirm results and to determine patterns of different snapshots of the *Cadm1* promoter, we have undertaken two treatment trials in some cell lines **(Figure S6)**. Here, we describe the collective *M.Sss*I maps found in the lung cancer cell lines with little or no *Cadm1* gene expression.

For fragment BFR, a stretch of at least three unmethylated CpGs were observed in many clones. These three sites at −944, −924, −903 CpGs, are located inside nuc 1 and thus, suggesting the occupancy of this nucleosome **(Figure S2)**. For fragment 1FR, although there were clones that displayed a stretch of at least three unmethylated CpGs to suggest nucleosome occupancy (nuc 2), most clones were highly methylated CpGs and the source of this methylation could be both endogenous and due to *M.Sss*I treatment **(Figure S3)**.

For fragment MFR1, most of the 98 clones were highly methylated, except for the two CpGs at −423 and −408. These two CpGs are within the predicted binding sequence of transcription factors (PPARg, ER), and are at the border of nuc 3 **(Figure S4)**. Protection of these two CpG sites could be both from transcription binding and nucleosome sliding. EMSA results suggested that PPARg bind *in vitro* to the *Cadm1* promoter with nuclear extracts from the lung cancer cell line (A2C12), but not in normal lung **(Figure S8)**, and could play a role in lung cancer.

For fragment MFRA, there were five clones among 86 clones with almost no methylation to suggest nucleosome occupancy (nuc 3) **(Figure S5)**. There were also individual CpG sites that exhibited low methylation, especially the Sp1 binding sites at −224 and −211, located at the border of a nucleosome (nuc 4) **(Figure S5)**. Thus, the protection in the lung cancer cell lines at −224 and −211 could be due to both Sp1 binding and nucleosome occupancy.

For fragment TSFR1, most of the 84 clones displayed long stretches of unmethylated CpGs to suggest high nucleosome occupancy (nuc 4 and nuc 5) around the TSS (**Figure 2**). One patch of protection was evident in the CpG sites −224 to −188; another patch was at −174 to −90. The binding sites for Sp1 and Zf5 are inside a patch of unmethylated CpGs indicating occlusion due to nucleosome occupancy. To support this assumption of a nucleosome occupancy, the same stretch of unmethylation was found after treating the chromatin of a lung cancer cell line (GA7) with *M.Cvi*PI (GpC methylase), and in which the methylation of CpG sites ( =  endogenous CpG methylation) was scored (data not shown).

#### Lung cancer cell line (A2C12) after treatment with 5-aza-dC

**Figure 3:**
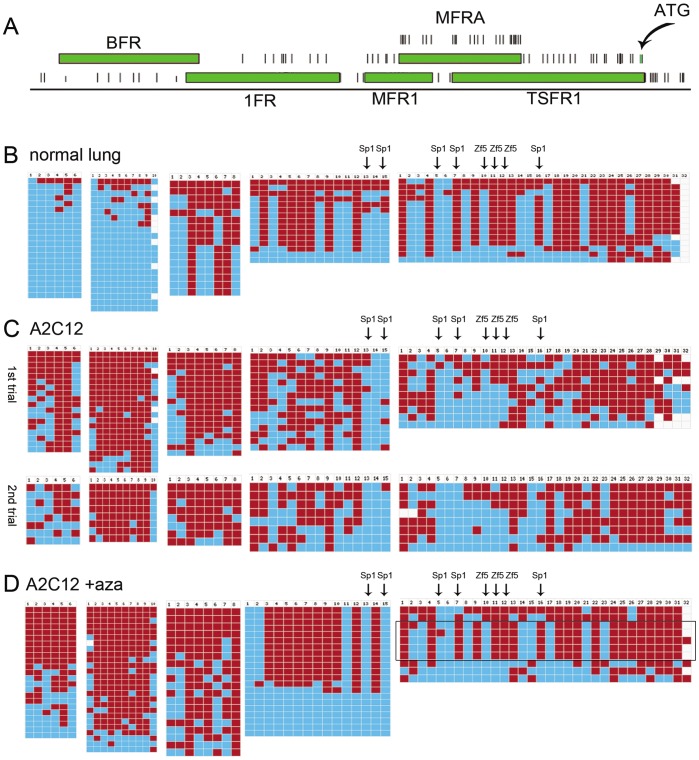
*M.SssI* maps in normal lung, and a lung cancer cell line (A2C12) with no *Cadm1* gene expression, before and after treatment with 5-aza-dC. (**A**) Location of the five fragments analyzed in the *Cadm1* promoter region that cover 69 CpGs −944 to +41, relative to the translation start site, ATG. CpGs are represented by stripes. (**B-D**) Methylation maps of normal lung and A2C12; blue boxes represents unmethylated CpGs ( = protected) while red boxes, methylated CpGs. The fragments are presented with respect to their location i.e. from BFR to TSFR1. The CpGs in the core sequence of Sp1 and Zf5 binding sites are indicated by arrows. (**D**) After 5-aza-dC treatment and slight gene re-expression, some clones resemble patterns found in normal lung (e.g. in TFSR1, enclosed), to suggest nucleosome remodeling (eviction) in gene expression.

The cell line A2C12 responds to 5-aza-dC treatment resulting in *Cadm1* re-expression. However, the degree of re-expression varies from treatment to treatment. We analyzed the *M.Sss*I map of 5-aza-dC-treated A2C12 which exhibited slight *Cadm1* gene re-expression and compared to non-treated A2C12 and normal lung (**Figure 3**). For fragment BFR, some clones now showed stretch of unmethylated CpGs, and this result suggested nucleosome occupancy (nuc 1). For fragment 1FR, three clones also showed stretches of unmethylated CpGs to indicate nucleosome occupancy (nuc 2).

In the next three fragments towards the TSS (MFR1, MFRA, TSFR1), some clones displayed patterns similar to normal lung, that is indicative of patterns associated with evicted nucleosomes and active transcription. For fragment MFR1, six clones resembled patterns of non-nucleosome occupancy and transcription factors binding as seen in normal lung. For fragment MFRA, there were only four patterns observed: 7/21 complete absence of methylation; 12/21 with the same pattern and clones appeared to be in the process of being remodelled or nucleosome being evicted; and 2/21 in between. The two Sp1 sites were occupied in all clones. Thus, Sp1 is binding in those clones with evicted nucleosomes, a finding that agrees well with our previous EMSA results [29]. For fragment TSFR1, several clones resembled pattern found in normal lung indicating absence of nucleosome but transcription factor binding. This result suggested chromatin remodeling after 5-aza-dC treatment.

#### Overall results M.SssI mapping

To summarize, *M.Sss*I mapping in normal lung supports the formation of at least five nucleosomes at the predicted positions along the promoter region of *Cadm1.* There were clones to show long stretches of unmethylated CpGs in these positions, especially in the two nucleosomes (nuc 1, nuc 2) upstream of TSS. The three closest nucleosomes (nuc 3, nuc 4, nuc 5) around the TSS and in the region where several predicted transcription factor binding sites are located, appeared to be absent in most clones. The patterns of these clones are suggestive of transcription factors binding or other proteins necessary for regulation. The binding sites for Sp1 and Zf5 were frequently unmethylated (protected) supporting further their role in the regulation of *Cadm1* expression. There were also other frequently protected sites that require further study.

Owing to endogenous CpG methylation, it was only possible to interpret the results in lung tumor and lung cancer cell lines from unmethylated CpGs (i.e. protected) after *M.Sss*I treatment. In lung tumor in which *Cadm1* was still expressed, there were stretches of unmethylated CpGs to support formation of three nucleosomes (nuc1, nuc 2, nuc 3). The two nucleosomes (nuc 4, nuc 5) most adjacent to the TSS appeared to be absent in analyzed clones. Notably, most clones displayed a common pattern involving methylation of few CpGs, and reminiscent of transcription factor binding. In the lung cancer cell lines with little or no *Cadm1* expression, results also suggested nucleosome formation (nuc 1 to nuc 5) in the predicted nucleotide positions. Stretches of unmethylated CpGs were frequent, especially those inside the nucleosomes. High nucleosome occupancy was observed especially in those three nucleosomes (nuc 3, nuc 4, nuc 5) closest to the TSS and where transcription factor binding sites that include Sp1 and Zf5 are located. In contrast to normal lung, there were no clones to show remodeling (or eviction) with respect to these three nucleosomes; thereby, transcription factor binding is blocked. After 5-aza-dC treatment of a lung cancer line (A2C12) that resulted in slight re-expression of *Cadm1,* remodeling was observed, and patterns found in normal lung became evident.

### Lung Cancer Cell Lines Show Differential Amplification Efficiency after MNase Digestion of Chromatin

**Figure 4:**
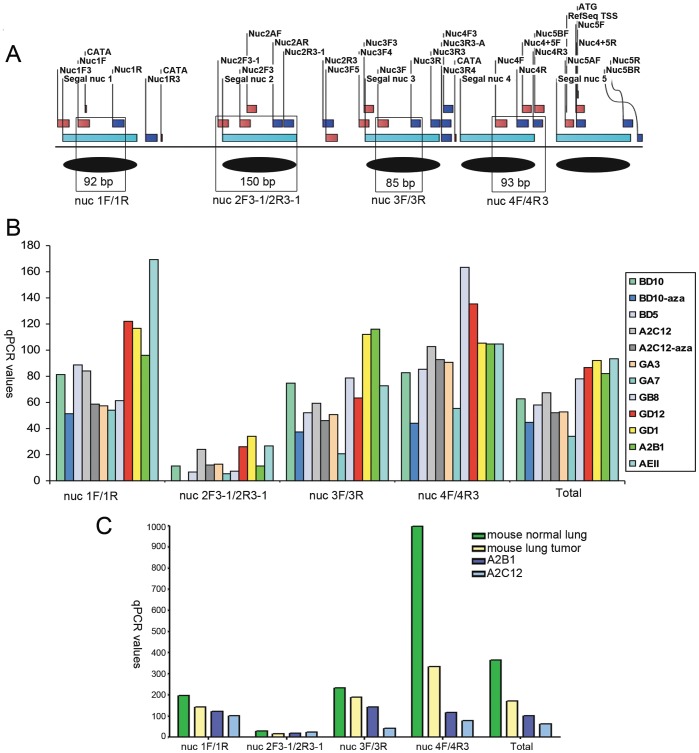
Chromatin analysis with micrococcal nuclease (MNase) to map nucleosome positions in *Cadm1* promoter region. **(A)** Position of five predicted nucleosomes using the Segal algorithm, the location of PCR primers used in amplifying fragments after digestion of chromatin with MNase, and MNase-preferred sites (CATA). Fragments that were also analyzed by quantitative-PCR are boxed. **(B)** Quantity of amplified fragments in different lung cancer cell lines, including two cell lines that were treated with 5-aza-dC, and a ’blind‘ control uncharacterized cell line (AEII) which does not express *Cadm1*. BD10-aza in nuc2F3-1/2R3-1 is a missing value. **(C)** Mouse normal lung, mouse lung tumor as compared to lung cancer cell lines, A2B1 and A2C12. The chromatin here analyzed for A2B1 and A2C12 are different from those in **(B)**.

Further to mapping with *M.Sss*I, we conducted chromatin analysis with micrococcal nuclease (MNase), an enzyme that preferentially cuts within nucleosomal linker regions and therefore useful in determining nucleosome positions [34]. MNase digestion of native chromatin in different samples resulted mainly in fragments of about 150–200 bp (mononucleosomes), but not in control ‘naked’ genomic DNA. Mononucleosomes were gel-isolated and interrogated by normal- and quantitative-PCR using a panel of PCR primers (see **Table S2**) amplifying within or at the left or right boundaries of predicted nucleosomes **(** **Figure 4** **, Figures S9, S10)**. The PCR products obtained with ‘middle’ (65–113 bp), ‘left’ (132–168 bp), and ‘right’ (93–222 bp) primers were verified by sequencing. The ‘naked’ genomic DNA was completely digested and no expected PCR products were obtained **(Figure** **S9)**.

We were able to amplify fragments for the five predicted nucleosomes in MNase-digested chromatin in normal lung, lung tumor and in the different lung cancer cell lines, suggesting nucleosomal nature of DNA (**Figure 4**). Primers designed to amplify products inside nucleosomes exhibited higher efficiency (banding intensity and/or qPCR values), than those primers that amplify bigger products and/or shifted to the left or right borders of nucleosomes. Furthermore, amplification efficiency differed among the lung cancer cell lines and was generally higher in cell lines which were less methylated and still expressed *Cadm1*. Such differences became more evident with the left or right border primers that in some cell lines amplification products were already absent. This result suggested differential nucleosome positioning among the different lung cancer cell lines. Overall, the highest amplification efficiency was observed in normal lung, then in lung tumor, and followed by the lung cancer cell lines.

To determine whether sequence alterations leading to variations in MNase digestions could be the cause for differential amplification efficiency, we sequenced the *Cadm1* promoter region in the different lung cancer cell lines. No sequence alterations were found. Since the use of native (non-fixed) chromatin may lead to sliding, we also compared PCR products from independent MNase digestions of native and formaldehyde-fixed chromatin from a lung cancer cell line (GD12, not shown). Expected PCR products were obtained. To test further the validity of our PCR protocol, we used primer pairs that span different nucleosomes or with bigger products. For instance, only few cell lines were positive to primer pair nuc 5 (nuc5BF/5BR) at 222 bp **(Figure S9)**. These control experiments thus support reliability of obtained results during MNase chromatin analysis.

### ChIP Corroborates High Nucleosome Occupancy Associated with Cadm1 Silencing in Lung Cancer Cells

We performed chromatin immunoprecipitation (ChIP) firstly, to confirm the nucleosomal nature of analyzed DNA fragments during *M.Sss*I mapping and MNase chromatin analysis, and secondly, to gain insights into the role of histone variants and modifications, which could affect nucleosome stability and positioning, in the transcriptional repression of *Cadm1* in the lung cancer cells. Essentially, ChIP experiments were undertaken on native chromatin (N-ChIP), in which chromatin was isolated using a buffer with 140 mM NaCl, and an MNase digestion giving mainly 150–200 bp fragments. Other salt conditions during chromatin preparation may influence nucleosome profile [35]. For the subsequent ChIP-PCR, we used the same panel of primers described previously in amplifying fragments from MNase-digested chromatin.

#### N-ChIP and X-ChIP with the canonical histone H2A

After N-ChIP with the canonical histone H2A, we could amplify most expected fragments from the five predicted nucleosomes in A2C12, a lung cancer line with no *Cadm1* gene expression **(Figure S11)**. Similar to MNase results, the intensity of amplification products inside nucleosomes was higher than those that included nucleosome borders, in agreement of sequences within the nucleosome core. This result evidencing nucleosome formation and occupancy at the given nucleotide positions has been confirmed in independent ChIP experiments. Indeed, to allay concerns of nucleosome re-arrangements during N-ChIP, we conducted a parallel experiment that included crosslinking of chromatin with formaldehyde (X-ChIP), different protocol for isolating and shearing of chromatin, and ChIP conditions (see Methods S1). X-ChIP with H2A, on a cell line (A2B1) with *Cadm1* gene expression, and three cell lines (GA7, GD12, A2C12), without *Cadm1* expression, yielded expected fragments confirming earlier results with N-ChIP **(Figure S11)**. Consistent of nucleosome depletion in gene expression, the quantity of amplified fragments in A2B1 was lesser than those cell lines without gene expression. Conversely, this result also suggested higher nucleosome occupancy associated with transcriptional repression of *Cadm1* in the lung cancer cell lines.

#### Comparison of H2A and H2A.Z nucleosomes in lung cancer cell lines with different gene expressions

Replacement of H2A with the histone variant H2A.Z could result in the sliding of nucleosomes to different positions, and would thereby affect gene expression [19]. To investigate such possibility, we compared ChIP results obtained with H2A and H2A.Z on different nucleosomes, in a cell line with *Cadm1* expression (A2B1) and a cell line without *Cadm1* expression (A2C12). N-ChIP and normal PCR using 2 µL of ChIP DNA showed amplification of both H2A and H2A.Z on nucleosomes around the TSS in the promoter region of *Cadm1* **(Figure S12B)**. Sequencing of these PCR products from the ChIP DNA with H2A and H2A.Z confirmed results. Furthermore, banding intensities showed overall that H2A was greater than H2A.Z in both cell lines, but the quantity of H2A and H2A.Z was higher in the cell line without *Cadm1* gene expression, suggesting higher nucleosome occupancy associated with silencing of the gene. Indeed, in some primer sets interrogating nucleosome borders, only H2A and H2A.Z from A2C12 could be amplified to imply also differential nucleosome positioning between A2B1 and A2C12. Furthermore, for extreme primer sets, e.g. nuc 4 (4F3/4R) with an expected product of 168 bp, only H2A from A2C12 gave a product, to likewise suggest different positioning between H2A and H2A.Z nucleosomes.

**Figure 5:**
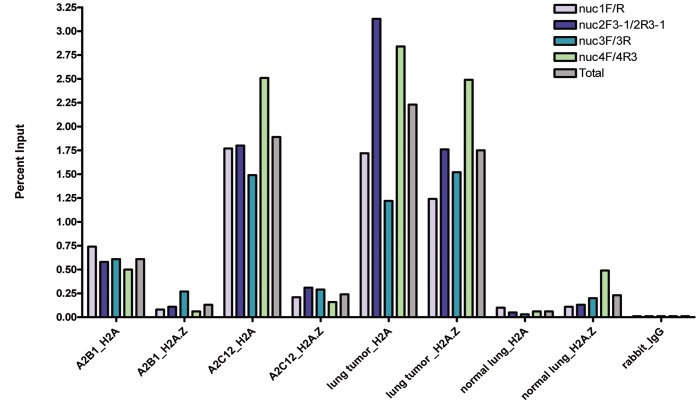
ChIP with H2A and H2A.Z in lung cancer cell lines, lung tumor, and normal lung. Results in analyzed nucleosomes are expressed as Percent Input using Ct values. The lung cancer line A2B1 still expresses *Cadm1*, while A2C12 does not. For quantitative PCR, 20 ng of ChIP DNA was used as template in all samples, including DNA obtained in normal rabbit IgG. The primer sets used and corresponding color coding are indicated on the uppermost right hand corner.

On the same ChIP experiment, we carried out quantitative PCR using four primer sets to assay four nucleosomes upstream of TSS using 20 ng of ChIP DNA from A2B1 and A2C12, and a dilution line established from an MNase-digested A2C12 chromatin. Overall, H2A values were greater than H2A.Z; and that A2B1 (H2A > H2A.Z) was lesser than A2C12 (H2A > H2A.Z) **(Figure S12C)**, in agreement with nucleosome depletion associated with gene expression. To confirm results, we performed independent N-ChIP with A2B1 vs. A2C12 and using Ct values as well as Percent Input normalization to interpret results (**Figure 5**). Altogether, in four independent experiments involving H2A and H2A.Z in A2B1 vs. A2C12, we found that H2A and H2A.Z were higher in A2C12 than in A2B1, a result suggestive of high nucleosome occupancy in transcriptional repression.

#### Comparison of H2A and H2A.Z nucleosomes in lung tumor

To determine the enrichments of H2A and H2A.Z nucleosomes in lung tumor, we conducted N-ChIP using chromatin isolated from two pooled solid lung tumors. *Cadm1* was still expressed in the tumors (data not shown). ChIP-PCR could amplify both H2A and H2A.Z at more or less same intensity in most all primers at predicted nucleosomes along the promoter region of *Cadm1* **(Figure S13)**. Quantitative PCR confirmed high enrichments of both H2A and H2A.Z in lung tumor. Overall H2A was greater than H2A.Z (**Figure 5**).

#### Comparison of H2A and H2A.Z nucleosomes in normal lung

We compared H2A and H2A.Z in normal lung chromatin isolated from seven pooled normal lungs of 11-month-old, non-transgenic female mice. Normal PCR with different primers trained at predicted nucleosomes showed the presence of both H2A and H2A.Z, but banding intensities in different primer pairs were higher in H2A.Z than in H2A **(Figure S14)**. Indeed, in some primer pairs trained at left or right boundaries of predicted nucleosomes, only H2A.Z could be amplified. A second independent N-ChIP similarly using chromatin isolated from seven pooled normal lungs and qPCR, confirmed that overall H2A.Z was higher than H2A in normal lung (**Figure 5**). ChIP values showed enrichment of H2A.Z at nuc 3 and nuc 4, the nucleosomes closest to the TSS (**Figure S14C**, Figure 5). This result found in normal lung differed from the lung cancer cell lines and lung tumors, in which overall H2A was greater than H2A.Z.

### Nucleosomes in the Cadm1 Promoter of Lung Cancer Cells are Enriched with Histone Variants and Histone Modifications

**Figure 6:**
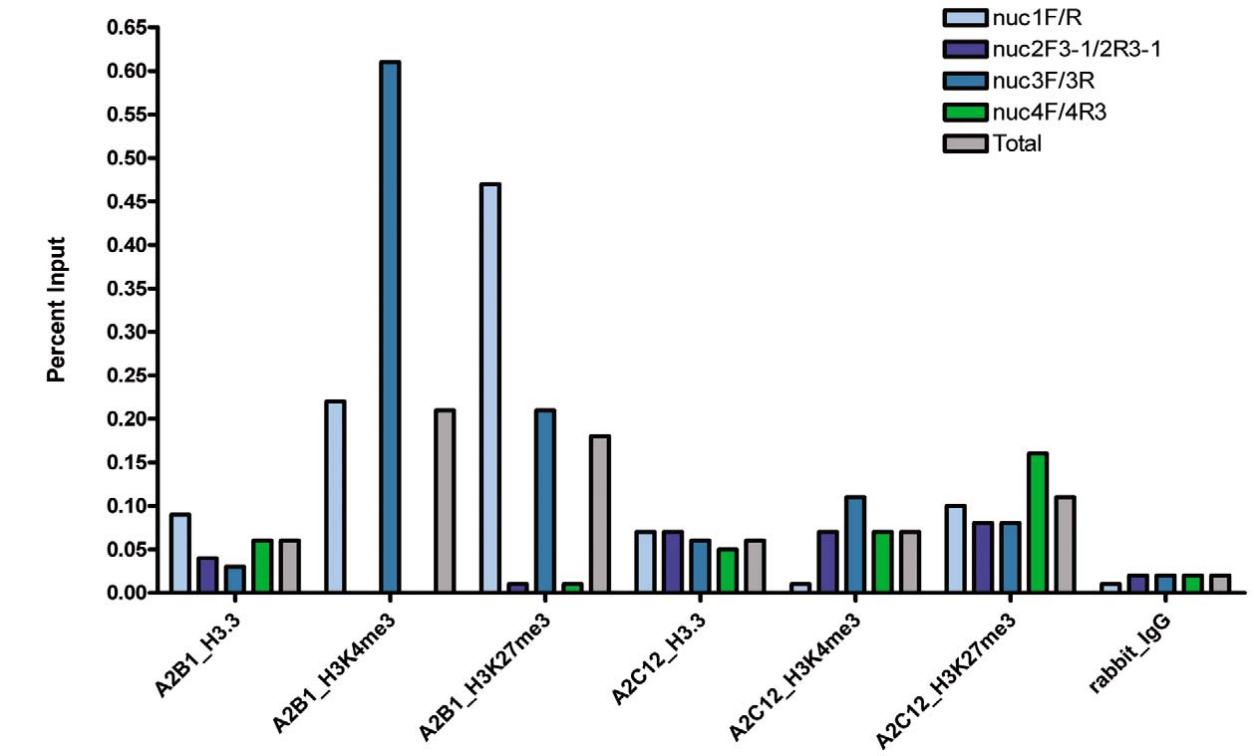
ChIP with histone variant (H3.3) and histone modifications (H3K4me3, H3K27me3) in lung cancer cells. ChIP experiments were undertaken in a lung cancer cell line with (A2B1) and without (A2C12) *Cadm1* gene expression. Results on different nucleosomes are expressed as Percent Input using Ct values. For quantitative PCR, template was adjusted to 20 ng for all samples, including DNA obtained in normal rabbit IgG. The primer sets used and corresponding color coding are indicated on the uppermost right hand corner.

Besides H2A.Z, we conducted ChIP with the histone variant H3.3, as well as the histone modifications H3K4me3 and H3K27me3, again on A2B1 (with *Cadm1* expression) and A2C12 (without *Cadm1* expression). ChIP results obtained using Ct values and Percent Input in analyzed nucleosomes in A2B1 vs. A2C12 are shown in **Figure 6**. For these experiments, H2A served as control for histone integrity. As for A2B1, it showed that enrichment of H3K4me3 and H3K27me3 was not in all nucleosomes. Indeed, the values for these histone modifications in nucleosomes 1 and 3 in A2B1 were higher than in A2C12.

**Figure 7:**
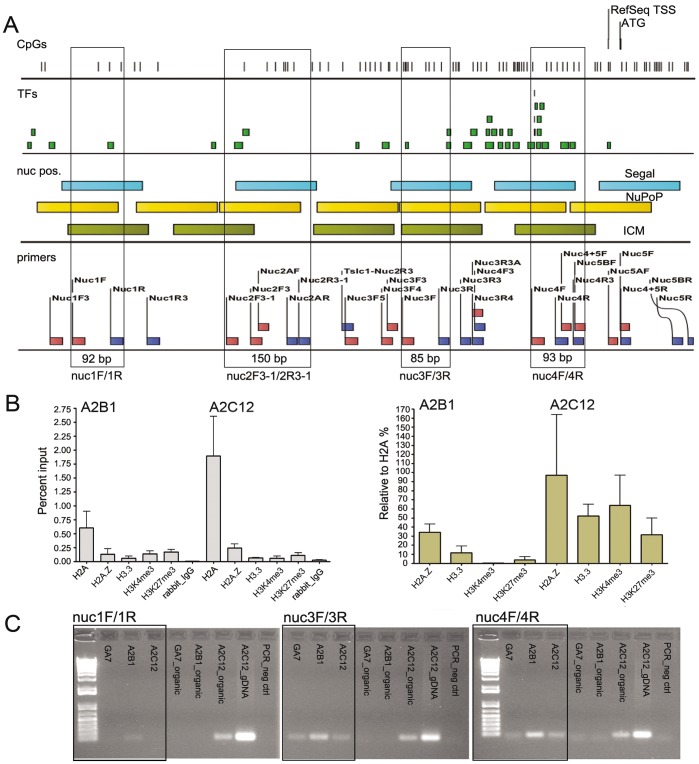
Chromatin status in repressed promoter of *Cadm1* in lung cancer cells. (**A**) Annotations of analyzed promoter region of *Cadm1*, showing location of CpGs, transcription factor binding sites, predicted nucleosome positions with different algorithms, and primers used in amplifying different fragments. Enclosed are positions of fragments analyzed by qPCR. (**B**) Comparison of total ChIPed DNA from different experiments with a canonical histone (H2A), histone variants (H3.3, H2A.Z) and histone modifications (H3K4me3, H3K27me3), in a lung cancer cell line with (A2B1) and without (A2C12) *Cadm1* gene expression. ChIP results are expressed as Percent Input using Ct values (left panel) and as further normalized relative to H2A (right panel). Normalization relative to H2A was undertaken at the level of nucleosomes in each experiment. (**C**) Amplified fragments in three different lung cancer cell lines after FAIRE method, i.e. formaldehyde crosslinking of chromatin and recovery of DNA fragments not bound by protein in the aqueous phase (boxed). The recovered DNA in the corresponding organic phase is also shown. In the aqueous phase (=open chromatin), banding intensity of amplified fragments in the cell line with *Cadm1* gene expression (A2B1) was higher than in the two cell lines without gene expression (GA7, A2C12). In the organic phase (=bound chromatin), fragments were only amplified in A2C12. Both positive control (A2C12 genomic DNA) and negative control (No DNA) for PCR are included during the analysis.

A summary of results from different ChIP experiments showed overall enrichments of histone variants and histone modifications of nucleosomes along the *Cadm1* promoter region in lung cancer cells in (**Figure 7B**, left panel). The corresponding results on different nucleosomes are shown in **Figure S15**. To determine enrichment relative to nucleosome density, we further normalized results relative to the canonical H2A, which was included in each ChIP experiment with the histone variants and modifications. This normalization method is assumed to correct for differences in ChIP signals that are caused by differences in the density of nucleosomes, rather than by changes in histone modification levels [36]. Furthermore, the rationale behind normalization relative to nucleosome density is that histone modifications can only be detected at a specific DNA sequence region if this region is also wrapped into nucleosomes. We found that A2C12 exhibited higher enrichments with respect to histone variants (H2A.Z, H3.3) and histone modifications (H3K4me3 and H3K27me3) than A2B1 (**Figure 7B**, right panel).

We also analyzed ChIP data on the basis of qPCR values obtained using 20 ng of ChIP DNA as template as well as calibration standards from a dilution series of gel-isolated MNase-digested chromatin of A2C12. Results confirmed overall that H2A was higher in A2C12 than A2B1, and also showed that H2A.Z, H3.3, H3K4me3, and H3K27me3 levels were higher in A2C12 than in A2B1 **(Figure S16)**. Furthermore, we have also undertaken normal PCR using 2 µl of ChIP DNA and a panel of 17 primer sets involving five different nucleosomes along the *Cadm1* promoter region. We observed higher banding intensities of PCR products obtained for H2A, H2A.Z, H3.3, H3K4me3, and H3K27me3 in A2C12 than in A2B1 (e.g. **Figure S12**). Altogether, ChIP data supported higher nucleosome occupancy, as well as higher enrichment of histone variants and histone modifications in A2C12 than in A2B1. The presence of such histone variants and histone modifications might also have an effect on nucleosome positioning, since the use of different primer pairs to interrogate nucleosomes especially on primers shifted to the left or right border of nucleosome, gave more PCR products in A2C12 than A2B1 (data not shown).

### FAIRE Regions are Higher in A2B1 than in A2C12

To determine whether there is indeed more open chromatin in A2B1 than A2C12, we adapted the FAIRE (formaldehyde-assisted isolation of regulatory elements) method [37] to our analysis. In this method, chromatin is crosslinked using formaldehyde, sonicated, and subjected to phenol-chloroform extraction. DNA fragments recovered in the aqueous phase (DNA not bound by protein) are then sequenced. Most enriched FAIRE regions were found near the transcription start sites (TSS), and overall there was a positive relationship between FAIRE signals and transcript levels. Using the FAIRE method and normal PCR using middle primers on nuc 1, nuc 3, and nuc 4, we found higher banding intensity in A2B1 than A2C12, and this increased towards the TSS, and thus suggestive of more open chromatin for A2B1 (**Figure 7C**). Consistent with this result, the corresponding organic phase (chromatin bound by protein) gave only products in A2C12.

## Discussion

The landscape of epigenetic silencing is complex, and although whole-genome analysis using next-generation technologies provides insights at the level of epigenome, there is still much to learn from functional dissection of silencing events at the promoter of single genes. In this study, we sought to understand the epigenetic silencing complexity in the promoter region of *Cadm1* in lung cancer progenitor cell lines established from single, spontaneously transformed lung tumors of *c-Myc* and *c-Raf* double-transgenic mice. Promoter hypermethylation in these cell lines correlates with transcriptional repression. We searched for additional epigenetic silencing events in the *Cadm1* promoter and compared results with normal lung and lung tumors. Using genomic sequence and bioinformatic tools, we predicted nucleosome positions and transcription factor binding sites along the *Cadm1* promoter. We carried out a rigorous single-molecule mapping of chromatin with the DNA methyltransferase *M.Sss*I to determine nucleosome occupancy and occlusion of transcription factor binding sites. With a panel of primers to interrogate the middle, left and right borders of predicted nucleosomes, we analyzed for differential nucleosome positioning in MNase-digested chromatin and ChIPed DNA with canonical histone H2A, histone variants H2A.Z and H3.3, as well as for histone modifications, H3K4me3 and H3K27me3. Overall, the present investigation defines a landscape of silencing characterized by high nucleosome occupancy, nucleosome sliding, DNA methylation, and enrichment of histone variants H3.3 and H2A.Z as well as histone modifications H3K4me3 and H3K27me3, which all colocalize at the same nucleosome positions.

Whether intrinsic DNA sequence preferences have a major role in determining the organization of nucleosomes *in vivo* is a subject of scientific debate [38], [39]). The discussion stems from contrasting findings in yeast after comparing positions of nucleosomes reconstituted *in vitro* to a map of *in vivo* locations. A recent study based on reconstituted nucleosome positioning in yeast also argued against a DNA-intrinsic or transcription-based mechanism for organizing nucleosomes around the 5′ ends of genes, but rather positioning appears to be driven by ATP-dependent activities that package nucleosomes against a 5′ barrier [40]. Our primary interest in using nucleosome positioning prediction algorithms based on genomic DNA sequence was to facilitate our analysis, and to streamline the design of primers to interrogate the chromatin state in normal and lung cancer cells. Nonetheless, we found that sequences within predicted nucleosome positions (i.e. nucleosome core sequences) could be amplified after MNase digestion and/or ChIP analysis with histone antibodies specifically H2A, using native or crosslinked chromatin. Furthermore, CpGs within these predicted nucleosomes showed protection after treatment of chromatin with the DNA methyltransferase, *M.Sss*I to suggest nucleosome occupancy.

Thus, the five analyzed nucleosomes as predicted using the algorithm from the Segal lab and described in several papers (e.g. [41]) are also formed *in vivo*. However, we observed possible alternative positioning of nucleosomes, in which we obtained in MNase-digested chromatin and ChIP-DNA a 125-bp or a 101-bp fragment that encompasses the supposed to be linker after nuc 4 and a part of nuc 5 (see **Figures S10, S11, S12, S13, S14**). Furthermore, differences in nucleosome borders in both normal and lung cancer cells were also found which may be attributed to the presence of modifying factors of nucleosome positions (see Introduction). Our results suggest that while DNA sequence influences the formation of nucleosome organization *in vivo*, sequence-dependent nucleosome positioning is probably not the sole determinant of chromatin organization.

Single-molecule chromatin mapping with *M.Sss*I showed high nucleosome occupancy in the lung cancer cell lines associated with promoter hypermethylation and transcriptional silencing. The nucleosomes in the region where transcription factor binding sites are located and near the transcriptional start site (TSS) were evicted after treatment with the demethylating agent, 5-aza-dC and was accompanied by slight re-expression of the gene. Furthermore, high nucleosome occupancy can be confirmed by the higher levels of ChIPed H2A in lung cancer cell lines with little or no *Cadm1* gene expression, than in a cell line that still expresses the gene, as obtained in native and in formaldehyde-crosslinked chromatin. These findings are similar to those obtained in the bidirectional *MLH1* promoter CpG island, in which three nucleosomes, almost completely absent from the three start sites in normal cells, were present on the methylated and silenced promoter of cancer cells [30]. They also observed that upon recovery of gene expression with 5-aza-dC, these nucleosomes were removed from the promoter molecules. High nucleosome occupancy suggests a tight or closed chromatin structure, and DNA methylation may have contributed to this configuration by increasing nucleosome compaction and rigidity [13].

The MNase chromatin analysis in different lung cancer cell lines, lung tumor and normal lung supports the formation of the analyzed nucleosomes along the promoter region of *Cadm1*. In agreement of sequences located at the nucleosome core, the quantity of PCR products of primers designed inside predicted nucleosomes was higher than those that interrogate nucleosome borders. Nonetheless, there were differences among the lung cancer cell lines. The quantity of qPCR product after MNase digestion seems to correlate with gene expression and the degree of methylation, i.e. those cell lines with gene expression have higher qPCR values than those repressed and highly methylated. Overall, highest amplification efficiency was observed in normal lung, then in lung tumor, and followed by the lung cancer cell lines. Similar findings have been demonstrated in maize gene, *ZmMI1* in which the correlation of methylation status with the nucleosomal structure was analyzed after MNase digestion of chromatin [42].

Chromatin analysis with MNase can reveal nucleosomal nature of DNA, but it has its own bias and thus requires control [43]. MNase has sequence preference; it cuts DNA primarily at runs of alternating dA and dT that are preceded by dG or dC. For example, CATA is a favored site, but a CATA site will be resistant to cleavage if located inside a nucleosome. Our analysis showed that MNase digestion of ‘naked’ genomic DNA did not yield the expected PCR products. In chromatin samples, a CATA site inside a nucleosome (nuc 1, **Figure S2**) did not lead to digestion suggestive of nucleosomal DNA being amplified. Furthermore, independent MNase digestions, including formaldehyde-crosslinked chromatin, in a lung cancer cell line, yielded expected PCR products (data not shown). Finally, ChIP results obtained with native chromatin coupled with MNase digestion could be confirmed with sonicated formaldehyde-crosslinked chromatin, and single-molecule mapping with DNA methyltransferase.

Notably, in normal lung H2A-containing nucleosomes especially near the transcription start site (TSS) were depleted. Similarly, the *M.Sss*I map of normal lung also showed absence of nucleosomes around the TSS in most clones, but instead transcription factors binding, e.g. Sp1. These observations provide further evidence that the region immediately upstream of the TSS of active genes is depleted of stable nucleosomes to allow binding of the transcriptional machinery to the DNA. This region is referred to as nucleosome-depleted region (NDR) or nucleosome-free region (NFR) in yeast promoters [44]. The NFR is defined as approximately 150 bp region that is devoid of nucleosomes and occurs at about 200 bp from the translation start site (ATG), and enriched with transcription factor binding sites and poly-deoxyadenosine or poly-deoxythymidine sequences. A question was raised regarding the term ‘NFR’ because certain types of nucleosomes were found unstable and underrepresented around the TSS, depending on the salt concentration used during chromatin isolation [45]. Our data presented here are based on chromatin isolated at high salt concentration (140 mM NaCl).

We also found in normal lung that H2A.Z binding correlated with active transcription, in which nucleosomes upstream and downstream of TSS in *Cadm1* promoter were enriched with H2A.Z. Indeed, H2A.Z was much higher than H2A in nucleosomes most adjacent to the TSS and ATG e.g. at nuc 3 and nuc 4 (−417 to –271 and −230 to −84 relative to ATG, respectively), and in the region where transcription factor binding sites are located. This finding in normal lung agrees with previous observations that H2A.Z is enriched in nucleosomes around the TSS of genes. In yeast, H2A.Z-nucleosomes flank one or both sides of the NFR that contains the TSS [46], while in human genome H2A.Z is highly enriched at promoter regions in both upstream and downstream of TSS [47]. Furthermore, in human T cells, H2A.Z-containing and modified nucleosomes are preferentially lost from the −1 nucleosome, relative to TSS [48].

We found further in normal lung that, H2A and H2A.Z-containing nucleosomes occupy the same DNA sequence, but H2A.Z appeared to differ from H2A as regards nucleosome borders. Using primer pairs that interrogate nucleosome boundaries, we obtained differential amplification of H2A and H2A.Z, in which certain primers gave only product for H2A.Z to imply different nucleosome positioning in the presence of H2A.Z. There is suggestion that replacement of H2A with H2A.Z in specific nucleosomes may result in the sliding of nucleosomes to different positions [20]. Indeed, there is evidence that incorporation of H2A.Z into promoter chromatin would allow nucleosomes to adopt preferential positions along the DNA translational axis, a condition that is permissive to the recruitment of the general transcriptional machinery [49]. Furthermore, it has been shown that H2A.Z-containing +1 nucleosomes of active genes shift upstream to occupy TSSs during mitosis, significantly reducing nucleosome-depleted region [50]. This mitotic shifting is specific to active genes that are silenced during mitosis and, thus, is not seen on promoters, which are silenced by methylation or mitotically expressed genes.

In lung tumor, the levels of H2A and H2A.Z did not vary much; nevertheless H2A was higher in quantity. In the two lung cancer cell lines with repressed *Cadm1* gene expression, H2A was also higher than H2A.Z. The higher values for H2A that correlated with transcriptional repression, suggest non-depletion of nucleosomes, and in agreement of a silent chromatin state. Interestingly, the lung cancer cell line (A2C12) without gene expression displayed higher values for both H2A and H2A.Z, than the cell line (A2B1) that still expresses the gene, to indicate certain depletion of nucleosomes in A2B1 than in A2C12. Indeed, the FAIRE results support more open chromatin for A2B1 than A2C12. Furthermore, primers to interrogate nucleosome borders likewise amplified H2A and H2A.Z fragments in A2C12, but no longer in A2B1 to reflect not only non-depletion of nucleosomes but altered nucleosome positioning as well. To summarize, H2A.Z-nucleosome occupancy was observed in both active and silent transcriptions, but enrichment levels was highest in normal lung and lowest in the lung cancer cell lines that displayed promoter hypermethylation.

Although H2A.Z has been extensively studied, its exact role in gene regulation remains unclear, being associated with both active and repressed states of gene expression. In yeast, H2A.Z was found to mark the 5′ ends of both active and inactive genes in euchromatin [46]. In ES cells, similar association of H2A.Z enrichment to both states of gene expression was likewise observed [51]. Genome-wide analysis of H2A.Z showed occupancy at promoters of a large set of silent developmental genes, in a manner similar to Polycomb group (PcG) proteins, which are known as transcriptional repressors. Conversely, H2A.Z enrichment was detected at active genes in multipotent neural precursors. Furthermore, in reconstituted nucleosomes, H2A.Z was shown to inhibit transcription [52]. To reconcile the positive and negative roles of histone H2A.Z in gene expression, and along with the observation that H2A.Z incorporation within a nucleosome leads to repositioning of a subset of nucleosomes to a position, it has been postulated that depending on where nucleosomes are repositioned, positive or negative effects on gene expression could be observed [53].

It has been demonstrated that the histone H2A.Z and DNA methylation are mutually antagonistic chromatin marks, in *A. thaliana* [54], and in puffer fish [55]. Similar relationship was also observed in mammals using a mouse B-cell lymphoma model, where chromatin states can be monitored during tumorigenesis [56]. H2A.Z and DNA methylation were found to be generally anti-correlated around TSS in both wild-type and Myc-transformed cells. Furthermore, there was progressive depletion of H2A.Z around TSS during Myc-induced transformation of pre-B cells and, subsequently during lymphomagenesis. In our study, this relationship seems also to hold true since H2A.Z occupancy was found highest in normal lung which did not display DNA methylation in the promoter region of *Cadm1*, and this became less in the hypermethylated lung cancer cell lines.

In the lung cancer cell lines, we found colocalization of two histone variants (H3.3 and H2A.Z) and histone modifications (H3K4me3 and H3K27me3) in the same nucleosome positions in the promoter region of *Cadm1*. This result may be a reflection of single histone variants or modifications affecting single nucleosomes within a cell line, but combinations in the same nucleosome within a cell are not unlikely. Indeed, different histone combinations can occur with structural or functional consequences. For instance, a single octameric nucleosome can contain two H2A.Z histones (homotypic) or one H2A.Z and one canonical H2A (heterotypic), and such homotypic nucleosomes were found to be enriched and heterotypic nucleosomes were depleted downstream of active promoters and intron-exon junctions [57]. H2A.Z and H3.3 double variant nucleosomes can also affect nucleosome positioning, either creating new positions or altering the relative occupancy of the existing nucleosome position space, while only H2A.Z-containing nucleosomes exhibited altered linker histone binding [58]. In human cells, the H2A.Z and H3.3 double variant-containing nucleosomes mark ‘nucleosome-free’ regions of active promoters as well as other regulatory regions, such as enhancer and insulators [35]. These double variants are unstable and are lost in the preparative methods usually used in studying nucleosome structure, and this instability facilitates the access of transcription factors to promoters and other regulatory sites in vivo. Moreover, H2A-Z containing promoters also contain mono, di, trimethylated K4H3, in which H2A.Z-deposition or H3K4me3 modification may facilitate eviction or repositioning in the promoter regions of the human genome [48].

Deregulation of H3K4me3 and H3K27me3, which are catalyzed by tri-thorax-group (trxG) proteins and polycomb-group (PcG) proteins, respectively is associated with cancer development [59]. Nucleosomes containing histone modification H3K4me3 have been associated with active transcription, while those with H3K27me3, with transcriptional repression. Thus, for us to observe enrichment or depletion concerning these opposing histone marks in lung cancer lines with different transcriptional status was not unexpected. However, H3K4me3 and H3K27me3 both colocalized, together with the histone variants H2A.Z and H3.3, in the same nucleosome positions. Nevertheless, in contrast to the lung cancer cell lines A2C12 (without *Cadm1* gene expression), only low levels of H3K4me3 and H3K27me3 were observed in A2B1 (with *Cadm1* gene expression), and when detected these could only be amplified in not all nucleosomes (i.e. so far, in nuc 1 and nuc 3 (−1011 to −865 and −417 to −271 relative to ATG, respectively).

The colocalization of H3K4me3 and H3K27me3 in the *Cadm1* promoter in lung cancer progenitor cells is similar to observations made in embryonic stem cells as will be described below, and such given dual marks is associated with epigenetic plasticity of these cells. Several genome-wide maps of H3K4me3 and H3K27me3 notably in embryonic stem (ES) cells provide evidence of genes exhibiting “bivalent domains” associated with both histone modifications [60]–[63]. These bivalent domains that combine both the “repressive” and “activating” modifications are associated with transcriptional repression, to poise genes prior to activation or to stably repress genes during differentiation.

For example, in mouse embryonic stem cells, neural progenitor cells and embryonic fibroblasts, the relative levels of H3K4me3 and H3K27me3 modifications in promoter regions can be used effectively to discriminate genes that are expressed, poised for expression, or stably repressed [61]. In human embryonic stem cells, colocalization of H3K4me3 and H3K27me3 on the same promoters was found to be a rule rather than an exception [62]. This bivalent histone modification was not restricted to early developmental genes in ES cells to keep cells poised for activation, but also to pluripotency-associated genes that become repressed during differentiation.

Bivalent configurations were also observed in T-cells. Global mapping of H3K4me3 and H3K27me3 in differentiating lineages of mouse CD4^+^ T cells revealed a broad spectrum of epigenetic modification states of genes, contributing to specificity as well as plasticity in lineage fate determination [64]. Among these include the marking of both H3K4me3 and H3K27me3 in the promoter regions of the genes encoding for transcription factors Tbx1 and Gata3 in non-expressing lineages. Similarly, genome-wide analysis of histone methylation H3K4me3 and H3K27me3 and expression profiles in naïve and memory CD8^+^ T cells showed that correlation exists between gene expression and the amounts of H3K4me3 (positive correlation) and H3K27me3 (negative correlation) across the gene body [65].

Indeed, there are studies documenting bivalent domains involving H3K4me3 and H3K27me3 in cancer cells and response to epigenetic drug treatments [66], [67]. For example, transcriptional repression of *DACT3,* which is an epigenetic regulator of Wnt/β-catenin signaling in colorectal cancer, was not associated with CpG promoter methylation, but the presence of the bivalent histone modifications H3K4me3 and H3K27me3 in colon cancer cells [66]. This repression could be reversed with the combined use of S-adenosylhomocysteine hydroxylase inhibitor 3-deazaneplanocin A (DZNep) and histone deacetylase inhibitor trichostatin A (TSA), but not the combination of DNMT inhibitor 5-AzaC and DZNep or TSA. Another study in colon cancer cells demonstrated that bivalent domains also mark the promoters of genes that become DNA methylated in adult tumor cells to enforce transcriptional silencing [67]. Analysis of neighboring genes, including many frequently silenced in colon cancer cells, in a chromosomal region at 5q35.2 spanning 1.25 Mb showed that inactive domains are defined by low transcriptional rates, promoter DNA methylation, and the presence of bivalent histone marks, H3K4me3 and H3K27me3. Transcriptional up-regulation accompanied by full or partial DNA demethylation was observed in genes containing bivalent domains and methylated promoter CpG islands after 5-aza-dC or combined 5-aza-dC/TSA treatments, but not TSA alone. Lastly, in addition to bivalent histone modifications of H3K4me3 and H3K27me3, frequent promoter colocalization of transcriptionally opposing bi-, tri, and tetra-valent histone marks has been demonstrated to confer microenvironment-responsive epigenetic plasticity to ovarian cancer cells [68].

Our study may provide some mechanistic insights for follow-up investigations towards the regulation of *Cadm1* and its role in lung cancer. We have shown colocalization of canonical histone H2A with histone variants (H2A.Z, H3.3), and histone modifications (H3K4me3, H3K27me3) in same DNA sequence, which likewise exhibited heavy CpG methylation. The lung cancer cell line which did not express *Cadm1* (A2C12) exhibited higher values for these epigenetic modifications, and most likely that their presence contributed jointly or in parallel in the silencing of the gene. Indeed, CpG methylation can have a distinct influence on protein binding when it is present within a nucleosomal background. SILAC nucleosome affinity purifications (SNAP) identified proteins whose binding to nucleosomes is regulated by CpG methylation and histone modification H3K4me3, H3K9me3, H3K27me3 or their combination [18]. Among these proteins include, for instance, the origin recognition complex (ORC), which was identified to be methylation-sensitive nucleosome interactor and recruited cooperatively by DNA and histone methylation. Their results also showed PAX6 to be nucleosome interactor as well in the presence of both methylated CpGs and histone modifications. PAX6 belongs to the paired-box (PAX) gene family of transcription factors involved in normal development and disease. PAX genes are frequently expressed in cancer, and that endogenous PAX gene expression is required for the growth and survival of cancer cells [69]. It is tempting to speculate about the role of PAX6 in the lung cancer line A2C12, and whether this factor was also recruited. In the *Cadm1* promoter, a putative PAX6 binding site is within a nucleosomal DNA (nuc 3, CpG site −350, relative to ATG). In A2C12, the nuc 3 region is enriched with histone modifications H3K4me3 and H3K27me3, and the CpGs are heavily methylated. In the *M.Sss*I map of normal lung in clones where no apparent nucleosomes were present, the CpG within the core binding site of PAX6 was not protected to suggest no binding occurred.

Furthermore, *M.Sss*I maps and EMSA experiments suggested that PPARg might be binding to the *Cadm1* promoter in a lung cancer cell line (A2C12), but not in normal lung. Mutated binding sequence or 100x competition with a normal probe abolished this binding. PPARg’s binding to the putative site was observed in A2C12 nuclear extracts, of both untreated and treated with 5-aza-2dC. Peroxisome proliferator-activated receptors (PPAR)-g belongs to the nuclear hormone receptor superfamily of ligand-dependent transcription factors and may be relevant for lung cancer therapy (see review, [70]). PPARg is expressed in human lung cancer cell lines (both SCLC and NSCLC), and its expression in lung cancer patients correlates with differentiation status and survival. Furthermore, PPARg ligands have been shown to inhibit tumor growth and progression in preclinical models of lung cancer, by modulating various cellular processes in cancer cells, stromal cells and tumor microenvironment, through PPARg crosstalk with other signaling pathways. Whether PPARg binding could have contributed to the regulation of *Cadm1* in A2C12 lung cancer cell line, or have implications at all in the use of PPARg ligands, remains to be explored. The PPARg putative binding site is located at the left border (adjacent) of a nucleosome (nuc 3) which can be easily influenced not only by DNA methylation but by nucleosome sliding as well.

Taken together, we have employed several approaches in dissecting epigenetic silencing complexity in the promoter region of the tumor suppressor gene *Cadm1* in mouse lung cancer progenitor cells. Knowledge gained would help understand how different epigenetic landscapes contribute to lung tumorigenesis and response to epigenetic drug treatments. First of all, the CpGs in the promoter region of *Cadm1* exhibited DNA methylation and this promoter hypermethylation correlated with transcriptional repression of the gene. DNA methylation was heterogeneous within and among the different lung cancer cell lines. Mapping of chromatin in single promoters revealed high nucleosome occupancy associated with silencing, indicative of a compact chromatin structure that is refractory to nucleosome remodelling and dynamism necessary for active transcription. Indeed, in contrast to normal lung, nucleosomes were present especially in regions where transcription factor binding sites are located. More important, although high nucleosome occupancy was a common characteristic of silent promoters, chromatin maps showed heterogeneity within and among the different lung cancer cell lines. Moreover, chromatin analysis with micrococcal nuclease (MNase) suggested differential nucleosome positioning in the lung cancer cell lines, and such has implications in the binding of transcription factors found at the boundaries of nucleosomes. Chromatin immunoprecipitation (ChIP) with histone variants and histone modifications also showed differences in nucleosome boundaries in two lung cancer lines that differed in *Cadm1* gene expression. In a lung cancer cell line with no *Cadm1* gene expression, there was colocalization of histone variants (H2A.Z, H3.3) that when present as double-variant affects nucleosome stability and positioning, as well as histone modifications in which one is an activating mark (H3K4me3) and the other is a repressive mark (H3K27me3) in the same nucleosome position. There is likelihood of several combinatorial possibilities that could affect nucleosome structure and positioning, which in turn have implications in the binding of transcriptional machinery and chromatin remodelling proteins to the DNA. The presence of both activating (H3K4me3) and repressing histone modifications (H3K27me3) known as ‘bivalent’ marks suggests stem-cell features of the lung cancer cells. In conclusion, there is complexity in the landscape of epigenetic silencing which is defined not by single but by the combinations of several epigenetic events, thereby rendering varying response to epigenetic drugs.

## Materials and Methods

### Lung Cancer Cell Lines, Lung Tumors and Normal Lungs

We analyzed chromatin from lung cancer cell lines, solid lung tumors, and normal lungs from mice. The 10 lung cancer cell lines of transgenic *c-Raf*/c*-Myc* mice have been described previously [29]. The lung tumors were of *c-Raf* transgenic mice [71], while the normal lungs of non-transgenic mice. Chromatin of each cell line was investigated separately, but pooled chromatin from 2–3 lung tumors, or 7–9 normal lungs were used for different investigations.

The transgenic mice were established many years ago according to an approved protocol (33-42502-02/548) by the Lower Saxony State Office for Consumer Protection and Food Safety (Germany). The lung cancer cell lines from the transgenic mice were also established according to an approved protocol (33-42502-02/548) by the Lower Saxony State Office for Consumer Protection and Food Safety (Germany). The normal lungs were from non-transgenic mice obtained from the Charles River Laboratories. Sectioning was undertaken by a trained scientist and registered at the regulatory office (33.42502/2) to carry-out animal experiments. The maintenance of animal models used in this study is carried out in strict accordance to regulations of care and use of laboratory animals by the same regulatory office.

### Bioinformatic Predictions

Different bioinformatic tools were utilized to predict nucleosome positions along the promoter region of *Cadm1*, which are based primarily on nucleosome positioning encoded in the DNA sequence. The online nucleosome prediction by genomic sequence (http://genie.weizmann.ac.il/software/nucleoprediction.html) from the Segal lab described in several papers [41], [72], [73] is based on the notion that DNA sequence is highly predictive of nucleosome positioning, and that certain sequences such as poly (dA:dT ) tracts are strongly disfavored by nucleosomes. The NuPoP: Nucleosome Positioning Prediction Engine (http://nucleosome.stats.northwestern.edu/) [74], predicts nucleosome positioning using a Hidden Markov Model by explicitly modeling the linker DNA length. The ICM Web (http://dna.ccs.tulane.edu/icm/) [75] allows the users to rapidly assess nucleosome stability and fold sequences of DNA into putative chromatin templates. Using TRANSFAC (http://www.gene-regulation.com/pub/databases.html), we analyzed putative binding sites of transcription factors in the promoter region of *Cadm1*.

### Primer Sequences

PCR primers to analyze the *Cadm1* promoter region in bisulfite-treated genomic DNA in mice were reported earlier [29]. PCR primers used during MNase and ChIP analyses are given in **Table S2**. These primers were designed based on predicted nucleosomes from the Segal lab algorithm. The PCR products of different primer combinations ranging from 66–222 bp, and location on the *Cadm1* promoter region are described in **Table S3**. Specificity of PCR primers were validated by sequencing of amplified fragments in mouse genomic DNA as well as in ChIP DNA, and 100% homology to a reference sequence (AC121870.2 within nt 161329–162348). The same primer pairs were also used to determine sequence alterations of the promoter region in different lung cancer cell lines.

### Gene Expression Analysis

Total RNA was isolated from frozen mouse lung tissues or cell lines with RNeasy Mini kit, and reverse transcription–PCR (RT-PCR) was undertaken with Omniscript RT kit (Qiagen). Semi-quantitative RT-PCR was performed using Thermostart *Taq* polymerase (ABgene) on T3 thermocyclers (Biometra), whereas quantitative RT-PCR on Light Cycler (Roche) using Absolute qPCR Sybr Green Capillary (ThermoFisher). Typically, 25 to 50 ng of cDNA were used for template. Reaction components and cycling variables were according to standard procedure.

### Analysis of Bisulfite-treated Genomic DNA

Genomic DNA was isolated in tissue samples and cell lines using Nucleo Spin Tissue (Macherey-Nagel). Bisulfite treatment was undertaken with EpiTect Bisulfite kit (Qiagen) using manufacturer’s instructions. Primers for methylation assays were designed with MethPrimer (http://www.urogene.org/methprimer/index1.html). PCR fragments were directly sequenced using BigDyeTerminator v3.1 kit and ABI 3100 Genetic Analyzer (Applied Biosystems), or PCR fragments were cloned using TOPO TA Cloning kit (Invitrogen) before sequencing. Sequences were analyzed using SeqMan (Lasergene 7.0) and confirmed by BISMA analysis (http://biochem.jacobs-university.de/BDPC). During BISMA scoring, the CpGs within the primer sequences (i.e.in fragments MFR1, MFRA, TSFR1) were not included.

### 5-Aza-2-deoxycytidine Treatment of Cells

Cells (1×10^6^) seeded in T25 cell culture flasks containing 5 mL of DMEM with 10% FCS, 2x L-glutamine, and 2x penicillin/streptomycin. Cells were cultured 48 h, treated with fresh 2 µmol/L 5-aza-2-deoxycytidine (5-aza-dC; Sigma) dissolved in medium for 3 d, and allowed to recover for 2 d.

### Chromatin Isolation

For lung cancer cell lines, approximately 1×10^6^ cells were grown 2–3 d until about 100% confluency, pelleted, and washed twice with 1 mL cold PBS. Cell pellet was resuspended completely in 300 µL lysis buffer (NPB: 10 mM Tris-HCl pH 7.4, 2 mM MgCl_2_, 140 mM NaCl, plus 0.5% Triton X-100), supplemented with protease inhibitors consisting of 40 mM beta-glycerophosphate, 4 mM pefabloc, 1 mM sodium orthovanadate, 1 mM DTT, and 1x Complete™ Protease Inhibitor Cocktail (Roche). After 10 min incubation and the control of nuclei quality by microscopy, homogenate was carefully layered onto 400 µL 1 V/V 50% sucrose/NPB bed, and centrifuged at 10 min 4°C 14000 rpm to pellet nuclei for further experiments.

For normal lungs and lung tumors, fresh tissues were weighed and cut into small pieces. Pre-chilled homogenization buffer (10 mL/g tissue) was added to the samples, and cells were disrupted using a Potter homogenizer. The homogenization buffer (2.2 M sucrose, 10% glycerine, 10 mM Hepes pH 7.6, 15 mM KCl, 1 mM EDTA) was supplemented with protease inhibitors consisting of 40 mM beta-glycerophosphate, 1 mM sodium orthovanadate, 1 mM DTT, 1x Complete™ Protease Inhibitor Cocktail (Roche), 0.15 mM spermine, and 0.5 mM spermidine. Homogenates were transferred into ultracentrifuge tubes and centrifuged for 60 min, 24000 rpm at 2°C (Beckmann Coulter Optima™ LE-80K, SW28.1 or SW32 Ti rotor). After aspirating most of the supernatant, 1 mL of NPB plus 0.5% Triton X-100 and protease inhibitors was added, transferred onto 500 µL 1 V/V 50% sucrose/NPB bed, and centrifuged at 10 min 4°C 14000 rpm to pellet nuclei for further experiments.

### M.SssI Treatment of Chromatin

The nuclei pellet from chromatin isolation was washed with 100 µL 1x *M.Sss*I buffer (NEB, 10 mM Tris–HCl pH 7.9, 50 mM NaCl, 10 mM MgCl_2_, 1 mM DTT). The nuclei pellet was incubated in 150 µL reaction volume containing 60 U *M.Sss*I, 160 µM S-adenosylmethionine (SAM) and 1x *M.Sss*I buffer, for 20 min at 37°C, centrifuged to remove supernatant, and proceeded immediately to DNA isolation with Nucleospin Tissue Kit (Macherey-Nagel). As ‘naked’ control, 1 µg mouse genomic DNA was treated with 60 U of *M.Sss*I. Genomic DNA was bisulfite-treated for methylation analysis.

### MNase Digestion of Chromatin

The nuclei pellet from chromatin isolation was washed with 100 µL 1x MNase buffer (NEB, 50 mM Tris-HCl 5 mM CaCl_2_). The nuclei pellet was incubated in 250 µL reaction volume containing 60 U MNase (NEB, 0.3 µL of 2000 Gel Units) 1x MNase buffer, and 1x BSA for 15 min at 25°C. The reaction was centrifuged to remove supernatant, and proceeded immediately to a modified DNA isolation with Nucleospin Tissue Kit (Macherey-Nagel). Briefly, pellet was resuspended in 200 µL Buffer T1. After adding 25 µL Proteinase K solution (20 µg/µL) and 200 µL Buffer B3, reaction was incubated at 70°C for at least 15 min. DNA was extracted with phenol-chloroform:isoamyl alcohol (25∶24∶1), dissolved in 100 µL Elution buffer (Macherey-Nagel), and incubated with 3 µL of RNase A (10 µg/µL) for 1 h 37°C. The resulting fragments of about 150–200 bp were gel-isolated, amplified with nucleosome-trained primers, or directly used for ChIP. As control, a parallel digestion of ‘naked’ genomic DNA was undertaken. As template, 2 µL of isolated DNA for normal PCR, while 20 ng for qPCR was used.

### Chromatin Immunoprecipitation (ChIP)

ChIP experiments on histones were mostly carried out with N-ChIP, which uses native chromatin. In contrast, X-ChIP uses chromatin in which DNA and proteins are crosslinked usually with formaldehyde. For the N-ChIP, chromatin from the lung cancer cell lines and lung tissues were isolated and digested with MNase resulting mostly in about 150–200 bp fragments. For ChIP, MNase digestion was stopped by adding 5 µL of 0.5M EDTA (10 mM final concentration). The steps for primary antibody-Dynabeads® coupling, binding of chromatin to the beads, and washing were essentially according to suggested protocol (MAGnify™ Chromatin Immunoprecipitation System, Invitrogen), and buffers [76]. After washing, bead pellets were resuspended in a final volume of 200 µL Buffer T1 (Macherey-Nagel) and proceeded to DNA isolation steps described under MNase digestion of chromatin. A parallel ChIP with formaldehyde-crosslinked chromatin was carried out with H2A on some lung cancer cell lines. The X-ChIP procedure was as essentially described previously [77] (see **Methods S1**).

### Antibodies

Histone antibodies were obtained from Abcam: H2A (ab18255), H2A.Z (ab4174, ab18263), H3.3 (ab62642), H3K4me3 (ab1012), and H3K27me3 (ab6002). The normal rabbit IgG antibody was from Sta. Cruz Biotechnology (sc-2027). Generally, 5 µg of antibody was used for each ChIP experiment.

### ChIP-PCR and Analysis of Data

Both normal PCR and quantitative PCR (qPCR) were undertaken to analyze ChIP-DNA. For normal PCR, template was 2–5 µL of 100 µL eluted ChIP-DNA. For qPCR, template (ChIP samples and Input DNA) was adjusted to 20 ng. When DNA was detected in IgG controls, 20 ng of DNA was also used for qPCR and included in the analysis. Calibration standard for qPCR consisted of a dilution series of gel-isolated MNase-digested chromatin from a lung cancer cell line (A2C12). At least three independent ChIP experiments were undertaken when feasible. The qPCR data are given as non-normalized values as obtained by fit point algorithm on the Light Cycler (Roche), and with adjusted PCR template of 20 ng for all samples, and/or Percent Input values based on 1% of starting chromatin and Ct values.

## Supporting Information

Figure S1 ***M.Sss*** **I maps in controls, normal lung, and lung tumor**. **(A)** Location of the five fragments analyzed in the *Cadm1* promoter region that cover 69 CpGs −944 to +41, relative to the translation start site, ATG. CpGs are represented by stripes. The maps **(B–E)** were obtained with BISMA (http://biochem.jacobs-university.de/BDPC/), where blue boxes representing unmethylated CpGs ( = protected) while red boxes, methylated CpGs. The fragments are presented with respect to their location i.e. from BFR to TSFR1. In lung tumors and lung cancer cell lines, CpG methylation could be endogenous and/or from the *M.Sss*I treatment. A2C12 is a lung cancer cell line that does not express *Cadm1* and showed prior CpG methylation. The CpGs in the core sequence of Sp1 and Zf5 binding sites are indicated by arrows.
(TIF)

Figure S2 ***M.Sss*** **I maps in normal lung, lung tumor and lung cancer cell lines in CpGs within the BFR fragment (255 bp, 6 CpGs −944 to −837).** **(A)** Annotation of the BFR fragment showing the CpGs, predicted nucleosomes with the Segal, ICM, NuPOP algorithms, and two MNase-preferred (CATA) restriction sites. The maps **(B–D)** were obtained with BISMA where blue boxes representing unmethylated CpGs ( = protected) while red boxes, methylated CpGs. In lung tumors and lung cancer cell lines, CpG methylation could be endogenous and/or from the *M.Sss*I treatment. **(B–C)** In normal lung and lung tumor, the CpGs within a predicted nucleosome (e.g. nuc 1) were unmethylated to suggest nucleosome occupancy. **(D)** The methylation patterns in 104 clones from 7 lung cancer cell lines with little or no *Cadm1* gene expression. Several clones likewise exhibited same stretch of unmethylated CpGs, to also suggest nucleosome occupancy.
(TIF)

Figure S3 ***M.Sss*** **I maps in normal lung, lung tumor and lung cancer cell lines in CpGs within the 1FR fragment (279 bp, 10 CpGs, −682 to −531.** **(A)** Annotation of the 1FR fragment showing the CpGs, predicted nucleosomes with the Segal, ICM, NuPOP algorithms, and putative binding sites of lung-specific transcription factors (GR, NKX2-1). The maps **(B–D)** were obtained with BISMA, where blue boxes representing unmethylated CpGs ( = protected) while red boxes, methylated CpGs. In lung tumors and lung cancer cell lines, CpG methylation could be endogenous and/or from the *M.Sss*I treatment. **(B–C)** In normal lung and lung tumor, several clones show a stretch of unmethylated CpGs within a predicted nucleosome (e.g. nuc 2) to suggest nucleosome occupancy. **(D)** The methylation patterns in 108 clones from 7 lung cancer cell lines with little or no *Cadm1* gene expression. Some clones exhibited same stretch of unmethylated CpGs, to also suggest nucleosome occupancy.
(TIF)

Figure S4 ***M.Sss*** **I maps in normal lung, lung tumor and lung cancer cell lines in CpGs within the MFR1 fragment (124 bp, 14 CpGs, −456 to −341).** **(A)** Annotation of the MFR1 fragment showing the CpGs, predicted nucleosomes with the Segal, ICM, NuPOP algorithms, and putative binding sites of transcription factors (SP3, PPARg, ER, ETF). The maps **(B–D)** were obtained with BISMA where blue boxes representing unmethylated CpGs ( = protected) while red boxes, methylated CpGs. In lung tumors and lung cancer cell lines, CpG methylation could be endogenous and/or from the *M.Sss*I treatment. Fragment MFR1 was amplified by methylation-specific primers (with 3 CpGs in both forward and reverse primers), and these CpGs were excluded during BISMA analysis. **(B)** In normal lung, no stretch of unmethylated CpGs was observed to suggest nucleosome occupancy. Specific CpG sites were, however, protected which may indicate possible transcription factor binding (e.g. PPARg, ER, and ETF). **(C–D)** Endogenous DNA methylation complicates interpretation of the patterns found in lung tumor and lung cancer cell lines. Unmethylated CpGs which fall in a predicted nucleosome (nuc 3) were, however, observed in the 98 clones from 7 lung cancer cell lines with little or no *Cadm1* gene expression.
(TIF)

Figure S5 ***M.Sss*** **I maps in normal lung, lung tumor and lung cancer cell lines in CpGs within the MFRA fragment (222 bp, 27 CpGs, −396 to −180).** **(A)** Annotation of the MFRA fragment showing the CpGs, predicted nucleosomes with the Segal, ICM, NuPOP algorithms, and putative binding sites of lung-specific transcription factors. The maps **(B–D)** were obtained with BISMA, where blue boxes representing unmethylated CpGs ( = protected) while red boxes, methylated CpGs. In lung tumors and lung cancer cell lines, CpG methylation could be endogenous and/or from the *M.Sss*I treatment. The CpG in the core sequence of two Sp1 sites are indicated by arrows. **(B)** In normal lung, DNA methylation patterns suggest absence of nucleosome occupancy and possible transcription-factor binding. But clones are also present with a stretch of unmethylated CpGs that are located in a predicted nucleosome (nuc 3). **(C–D)** Endogenous DNA methylation complicates interpretation of the patterns found in lung tumor and lung cancer cell lines, but clones are present with a stretch of unmethylated CpGs that are located in a predicted nucleosome (nuc 3). In the 86 clones from 7 lung cancer cell lines with little or no *Cadm1* gene expression, the CpGs in the Sp1 binding sites at −224 and −211 were mostly unmethylated, which could be both due to Sp1 binding and nucleosome sliding.
(TIF)

Figure S6 ***M.Sss*** **I maps in first and second trials in three lung cancer cell lines (B3, A2B1 and BD10).** **(A)** Location of the five fragments analyzed in the *Cadm1* promoter region that cover 69 CpGs −944 to +41, relative to the translation start site, ATG. The maps **(B–D)** were obtained with BISMA, where blue boxes representing unmethylated CpGs ( = protected) while red boxes, methylated CpGs. The fragments are presented with respect to their location i.e. from BFR to TSFR1. In the lung cancer cell lines, CpG methylation could be endogenous and/or from the *M.Sss*I treatment. The lung cancer cell lines (B3, A2B1 and BD10) still express *Cadm1*, with BD10 the lowest. The CpGs in the core sequence of Sp1 and Zf5 binding sites are indicated by arrows.
(TIF)

Figure S7 ***M.Sss*** **I maps in six lung cancer cell lines with little or no** ***Cadm1*** **gene expression.** **(A)** Location of the five fragments analyzed in the *Cadm1* promoter region that cover 69 CpGs −944 to +41, relative to the translation start site, ATG. CpGs are represented by stripes. **(B)** Methylation maps were obtained with BISMA, where blue boxes representing unmethylated CpGs ( = protected) while red boxes, methylated CpGs. The fragments are presented with respect to their location i.e. from BFR to TSFR1. In the lung cancer cell lines, CpG methylation could be endogenous and/or from the *M.Sss*I treatment. The CpGs in the core sequence of Sp1 and Zf5 binding sites are indicated by arrows.
(TIF)

Figure S8 **EMSA experiments with PPARg.** **(A)** The predicted PPARg binding sequence in the *Cadm1* promoter was used as a probe in nuclear extracts from normal lung, a lung cancer cell line with no *Cadm1* gene expression (A2C12), the cell line A2C12 treated with 5-aza-2′-deoxycytidine, and a Caco cell line used as control. No binding was observed in normal lung and in the Caco cell line. In A2C12, where binding occurred, no clear supershift was observed after addition of PPARg antibody, but the band (arrow) became weak as compared to the sample without the antibody. **(B)** Mutated PPARg core sequence led to abolition of binding. **(C)** 100x competition with the wild type probe also abolished binding. Negative controls were A2C12 nuclear extracts with no added probes. EMSA probes: WT_F 5′ tctcgcggtcagactctccgacca 3′, WT_R 5′ tggtcggagagtctgaccgcgaga 3′, MUT_F 5′tctcgctggctgactctccgacca 3′, MUT_R 5′ tggtcggagagtcagccagcgaga 3′. Antibody PPARgamma (H-100) sc-7196X Sta Cruz Biotechnology.
(TIF)

Figure S9 **Chromatin analysis with micrococcal nuclease (MNase** ***)*** **in mouse lung cancer cell lines.** **(A)** DNA fragments after MNase digestion of chromatin and ‘naked’ genomic DNA in a lung cancer cell line (A2C12). No PCR product was obtained in the ‘naked’ genomic DNA (right panel). **(B)** Normal PCR products with different primers designed on predicted nucleosomes and 2 µL of MNase-digested chromatin as template in the lung cancer cell lines. The samples were analyzed and loaded onto the gel in the same order as given above. Shown are also two cell lines that were treated with 5-aza-dC, and a ‘blind’ control uncharacterized cell line (AEII) which does not express *Cadm1*. On the upper left corner of each gel are the primer pairs and the size of products. The quantity of PCR products of ‘middle’ primers (middle panel) was higher than those in which one primer is moved towards the left or right border of a nucleosome (left and right panels, respectively). Undigested genomic DNA from a lung cancer cell line (GA3) was used as positive control.
(TIF)

Figure S10 **Chromatin analysis with micrococcal nuclease (MNase) in mouse normal lung. (A)** Position of five predicted nucleosomes obtained with the Segal lab algorithm, the location of PCR primers used in amplifying fragments after digestion of chromatin with MNase, and MNase-preferred sites (CATA). **(B)** DNA fragment after MNase digestion of chromatin from seven pooled normal lungs. The quality and concentration of DNA was checked on 1% ethidium bromide gel before performing PCR in **(C)**. Normal PCR products with different primers designed on predicted nucleosomes and 2 µL of MNase-digested chromatin as template. 1: normal lung chromatin, 2: undigested genomic DNA from a lung cancer cell line (A2C12) as positive control, 3: PCR negative control. The primer pair nuc5AF/5R amplified an additional fragment in undigested genomic DNA.
(TIF)

Figure S11 **ChIP experiments with H2A using native and crosslinked chromatin in lung cancer cell lines.** **(A)** Position of predicted nucleosomes obtained by different algorithms, location of primers and examples of product size of amplified fragments. **(B)** Different products from normal PCR and 2 µL of ChIP DNA as template following N-ChIP in A2C12. Loaded onto gel from left in each primer pair after the size marker (Kb ladder), **1–2:** ChIP with different chromatin isolation batches, **3:** gel-isolated MNase-digested chromatin, **4:** undigested genomic DNA control. **5:** PCR negative control. The primer pair nuc5AF/5AR amplifies an additional bigger fragment in undigested genomic DNA. **(C)** An independent N-ChIP experiment with A2C12. **(D)** X-ChIP with different cell lines showing amplification of same fragments in selected primer pairs. Less PCR product was obtained with A2B1 which still expresses *Cadm1*, as compared to those without expression.
(TIF)

Figure S12 **N-ChIP experiments with H2A and H2A.Z in lung cancer cell lines (A2B1 vs. A2C12).** **(A)** Position of predicted nucleosomes obtained by different algorithms, location of primers and examples of product size of amplified fragments. **(B)** Different products from normal PCR and 2 µl of ChIP DNA as template following ChIP with A2B1 and A2C12. Samples were loaded onto gel as shown for the primer pair nuc1F/1R. The primer pair nuc5AF/5AR amplifies an additional bigger fragment in undigested genomic DNA. Some products are already absent in A2B1. **(C)** Corresponding qPCR with selected primers, using 20 ng of ChIP DNA as template; results are raw measurements.
(TIF)

Figure S13 **N-ChIP experiments with H2A and H2A.Z in mouse lung tumor.** **(A)** Position of predicted nucleosomes obtained by different algorithms, location of primers and examples of product size of amplified fragments. **(B)** Different products from normal PCR and 2 µL of ChIP DNA as template following ChIP with lung tumor. Samples were loaded onto gel as shown for the primer pair nuc1F/1R. The primer pair nuc5AF/5AR amplifies an additional bigger fragment in undigested genomic DNA. **(C)** Corresponding qPCR with selected primers, using 20 ng of ChIP DNA as template.
(TIF)

Figure S14 **N-ChIP experiments with H2A and H2A.Z in mouse normal lung.** **(A)** Position of predicted nucleosomes obtained by different algorithms, location of primers and examples of product size of amplified fragments. **(B)** Different products from normal PCR and 2 µL of ChIP DNA as template following ChIP with chromatin isolated from seven pooled normal lungs. In this experiment, the parallel MNase-digested chromatin used as control was not optimal. Nonetheless, the Input DNA is basically the same as the MNase control. Samples were loaded onto gel as shown for the primer pair nuc1F/1R. Some primer pairs gave weak products even in the positive control (undigested genomic DNA A2C12). The primer pair nuc5AF/5AR amplifies an additional bigger fragment in undigested genomic DNA. **(C)** Quantitative PCR with selected primers, using 20 ng of ChIP DNA as template. The ChIP DNA as measured by the qPCR was obtained from an independent experiment.
(TIF)

Figure S15 **Comparison of chromatin status between A2B1 and A2C12 (A)** Position of predicted nucleosomes obtained by different algorithms, location of primers and examples of product size of amplified fragments. **(B)** Comparison of ChIP DNA with a canonical histone (H2A), histone variants (H3.3, H2A.Z) and histone modifications (H3K4me3, H3K27me3), in different fragments analyzed by qPCR. ChIP results are expressed as Percent Input using Ct values. In A2B1, not all fragments in ChIP with H3K4me3 and H3K27me3 could be amplified.
(TIF)

Figure S16 **Comparison of different histones between A2B1 and A2C12 (A–B).** Examples of ChIP experiments carried out on the same day in A2B1 and A2C12 in different fragments analyzed by qPCR. The result is based on qPCR values obtained with 20 ng of ChIP DNA as template and using calibration standards of a dilution series of gel-isolated MNase-digested chromatin of A2C12.
(TIF)

Table S1 **Amplified fragments in the** ***Cadm1*** **promoter region to analyze CpG methylation in bisulfite-treated genomic DNA.**
(DOC)

Table S2 **Primer sequences used during MNase and ChIP experiments to interrogate nucleosome positioning in the promoter region of mouse** ***Cadm1*** **gene.**
(DOC)

Table S3 **Primer combinations and products used during MNase and ChIP experiments to interrogate nucleosome positioning in the promoter region of mouse** ***Cadm1*** **gene.**
(DOC)

Methods S1 **Chromatin immunoprecipitation (ChIP): X-ChIP.**
(DOC)
