# Supplementary figures and images for: Dissecting Epigenetic Silencing Complexity in the Mouse Lung Cancer Suppressor Gene Cadm1
Source: PLoS One. 2012 Jun 6;7(6):e38531. doi: 10.1371/journal.pone.0038531 (PMC3368868; doi:10.1371/journal.pone.0038531)

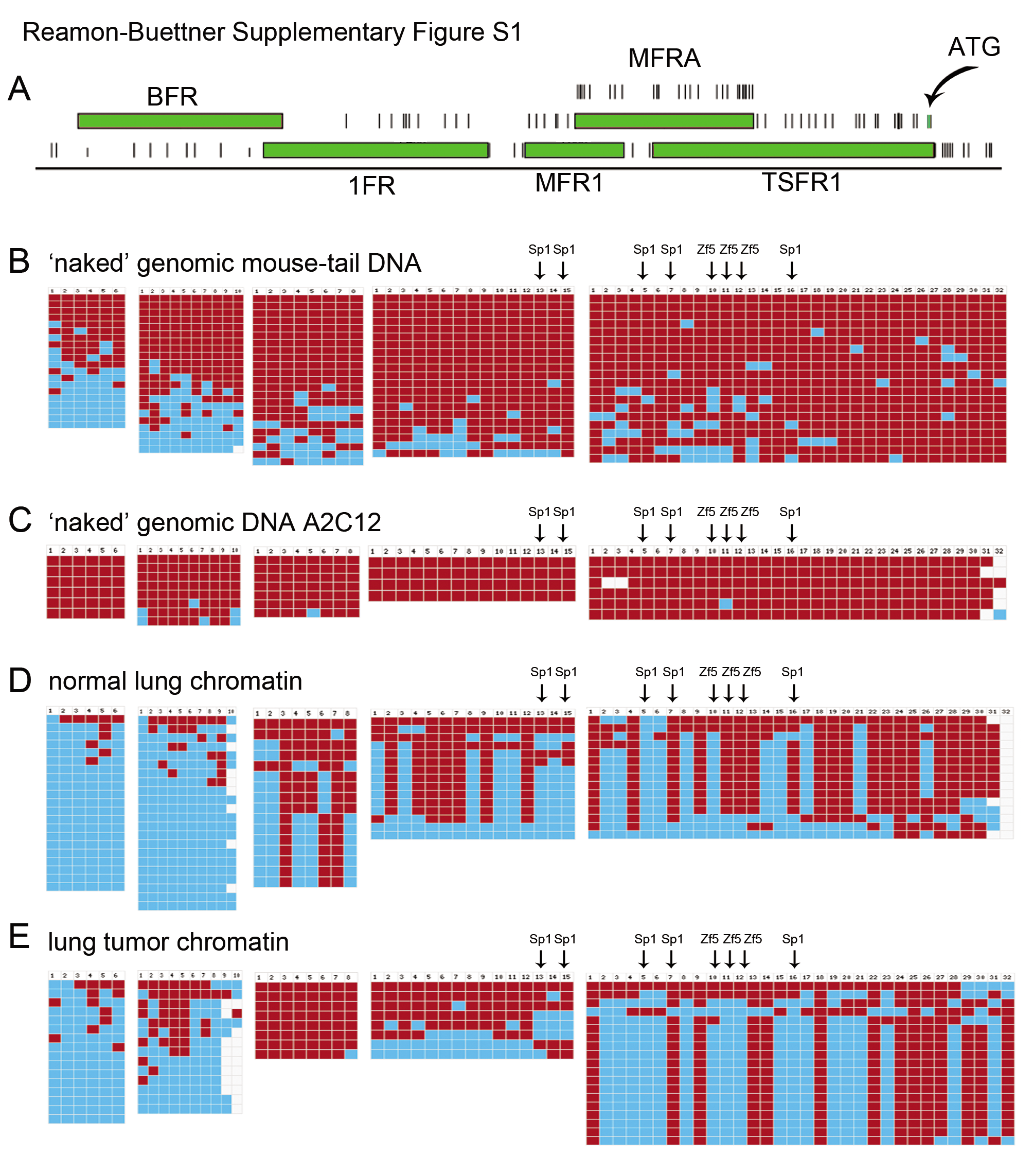

Supplement: Figure S1 — M.Sss I maps in controls, normal lung, and lung tumor. (A) Location of the five fragments analyzed in the Cadm1 promoter region that cover 69 CpGs −944 to +41, relative to the translation start site, ATG. CpGs are represented by stripes. The maps (B–E) were obtained with BISMA (http://biochem.jacobs-university.de/BDPC/), where blue boxes representing unmethylated CpGs ( = protected) while red boxes, methylated CpGs. The fragments are presented with respect to their location i.e. from BFR to TSFR1. In lung tumors and lung cancer cell lines, CpG methylation could be endogenous and/or from the M.SssI treatment. A2C12 is a lung cancer cell line that does not express Cadm1 and showed prior CpG methylation. The CpGs in the core sequence of Sp1 and Zf5 binding sites are indicated by arrows. (TIF) [file pone.0038531.s001.tif]

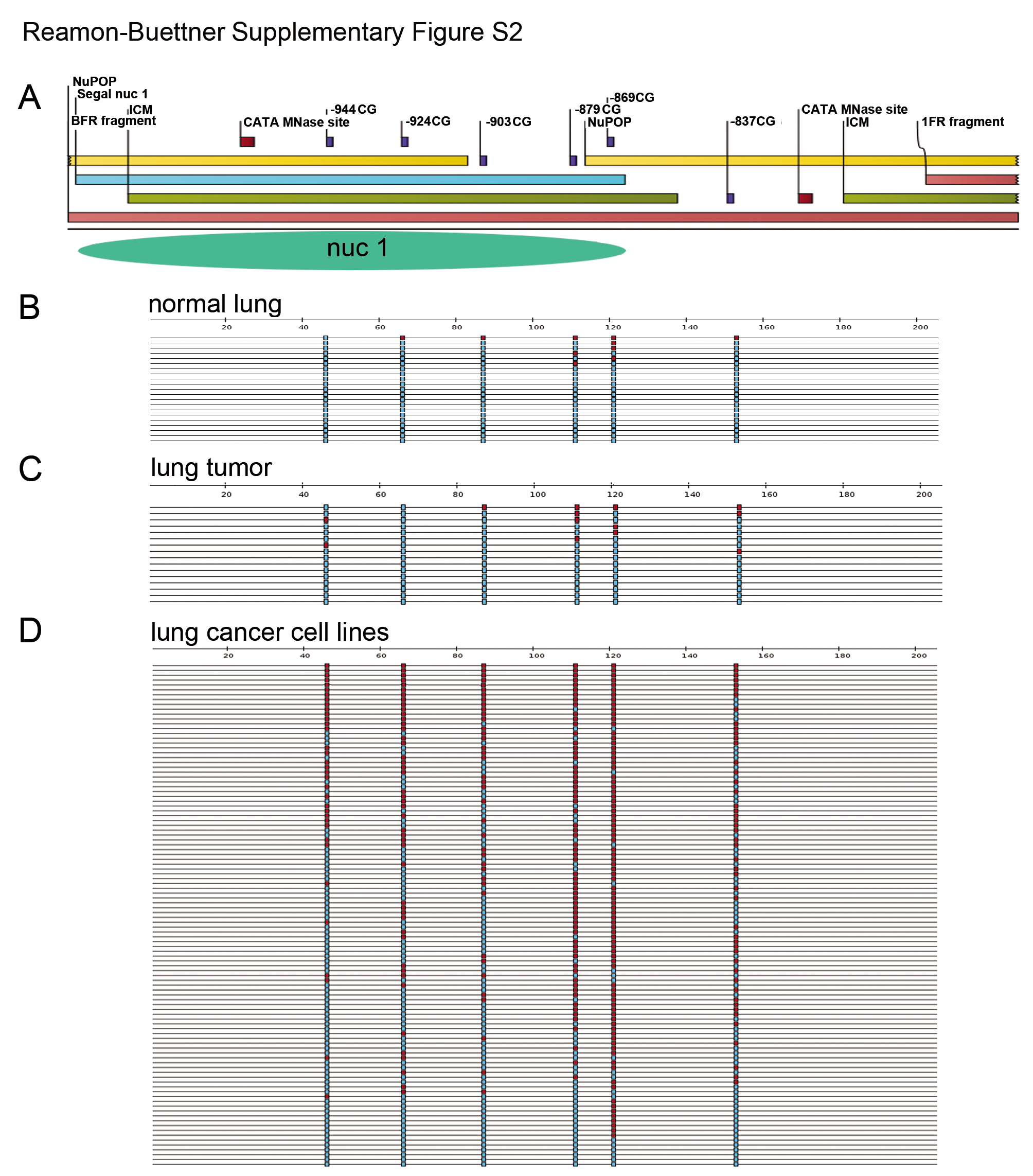

Supplement: Figure S2 — M.Sss I maps in normal lung, lung tumor and lung cancer cell lines in CpGs within the BFR fragment (255 bp, 6 CpGs −944 to −837). (A) Annotation of the BFR fragment showing the CpGs, predicted nucleosomes with the Segal, ICM, NuPOP algorithms, and two MNase-preferred (CATA) restriction sites. The maps (B–D) were obtained with BISMA where blue boxes representing unmethylated CpGs ( = protected) while red boxes, methylated CpGs. In lung tumors and lung cancer cell lines, CpG methylation could be endogenous and/or from the M.SssI treatment. (B–C) In normal lung and lung tumor, the CpGs within a predicted nucleosome (e.g. nuc 1) were unmethylated to suggest nucleosome occupancy. (D) The methylation patterns in 104 clones from 7 lung cancer cell lines with little or no Cadm1 gene expression. Several clones likewise exhibited same stretch of unmethylated CpGs, to also suggest nucleosome occupancy. (TIF) [file pone.0038531.s002.tif]

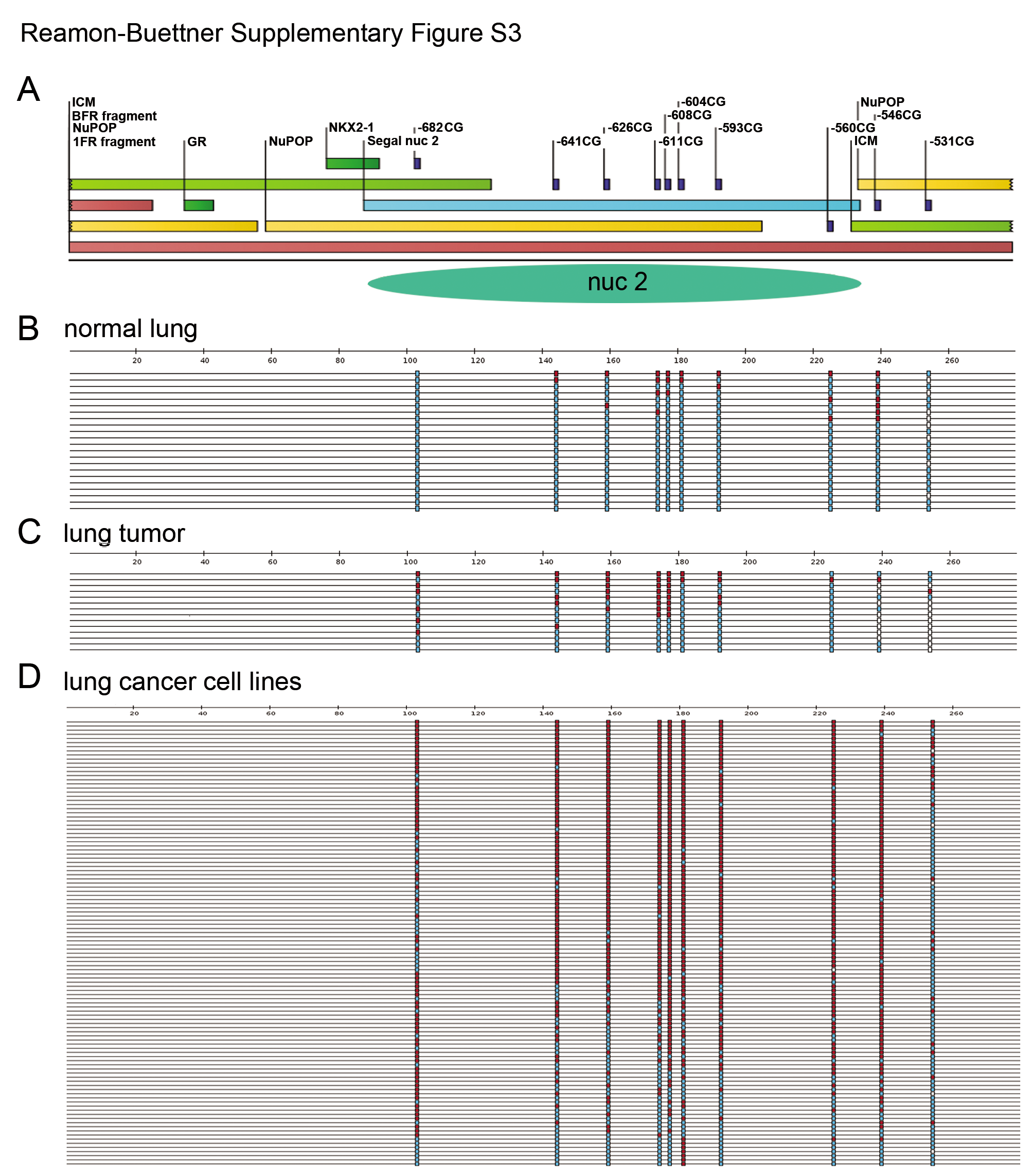

Supplement: Figure S3 — M.Sss I maps in normal lung, lung tumor and lung cancer cell lines in CpGs within the 1FR fragment (279 bp, 10 CpGs, −682 to −531. (A) Annotation of the 1FR fragment showing the CpGs, predicted nucleosomes with the Segal, ICM, NuPOP algorithms, and putative binding sites of lung-specific transcription factors (GR, NKX2-1). The maps (B–D) were obtained with BISMA, where blue boxes representing unmethylated CpGs ( = protected) while red boxes, methylated CpGs. In lung tumors and lung cancer cell lines, CpG methylation could be endogenous and/or from the M.SssI treatment. (B–C) In normal lung and lung tumor, several clones show a stretch of unmethylated CpGs within a predicted nucleosome (e.g. nuc 2) to suggest nucleosome occupancy. (D) The methylation patterns in 108 clones from 7 lung cancer cell lines with little or no Cadm1 gene expression. Some clones exhibited same stretch of unmethylated CpGs, to also suggest nucleosome occupancy. (TIF) [file pone.0038531.s003.tif]

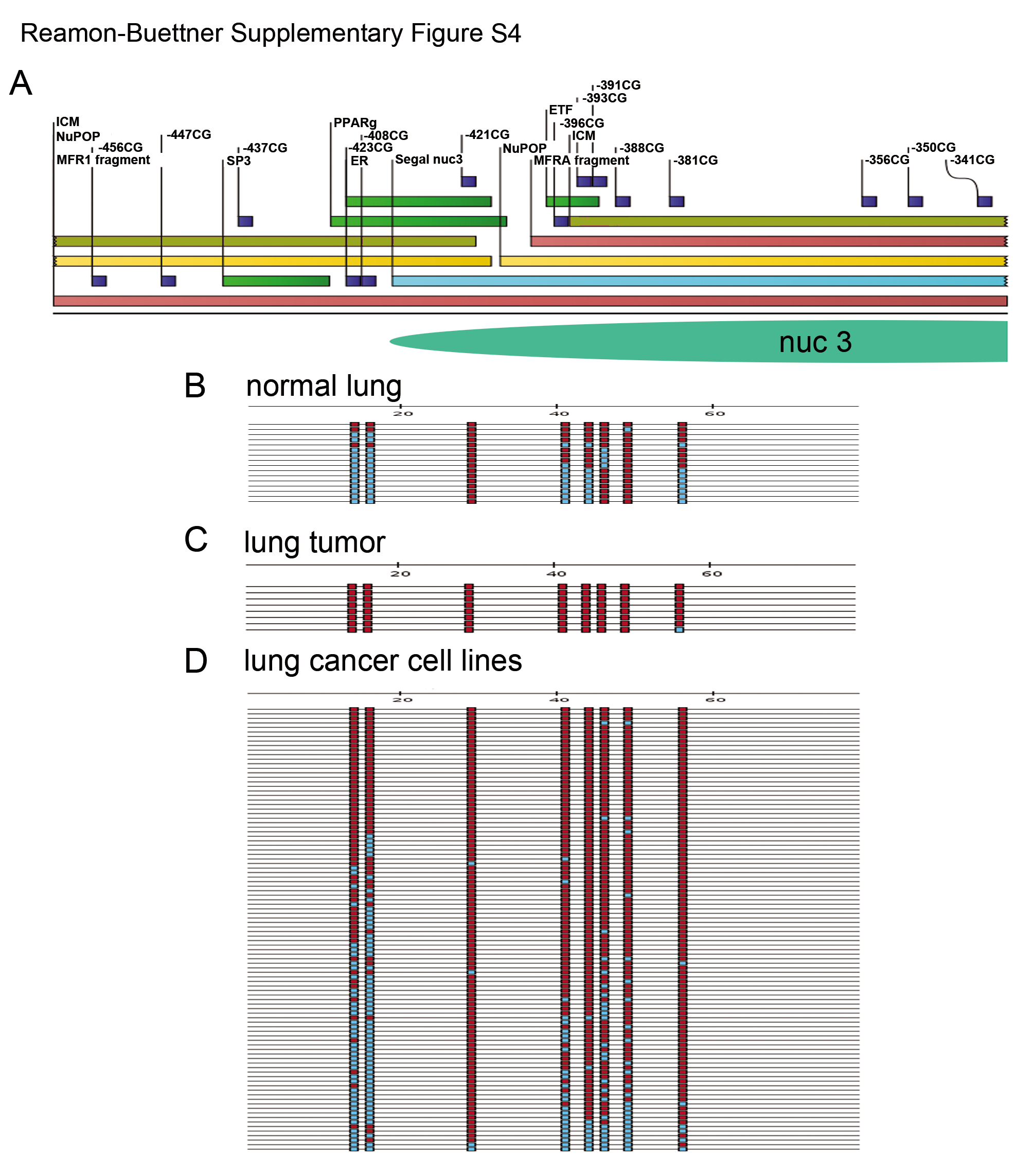

Supplement: Figure S4 — M.Sss I maps in normal lung, lung tumor and lung cancer cell lines in CpGs within the MFR1 fragment (124 bp, 14 CpGs, −456 to −341). (A) Annotation of the MFR1 fragment showing the CpGs, predicted nucleosomes with the Segal, ICM, NuPOP algorithms, and putative binding sites of transcription factors (SP3, PPARg, ER, ETF). The maps (B–D) were obtained with BISMA where blue boxes representing unmethylated CpGs ( = protected) while red boxes, methylated CpGs. In lung tumors and lung cancer cell lines, CpG methylation could be endogenous and/or from the M.SssI treatment. Fragment MFR1 was amplified by methylation-specific primers (with 3 CpGs in both forward and reverse primers), and these CpGs were excluded during BISMA analysis. (B) In normal lung, no stretch of unmethylated CpGs was observed to suggest nucleosome occupancy. Specific CpG sites were, however, protected which may indicate possible transcription factor binding (e.g. PPARg, ER, and ETF). (C–D) Endogenous DNA methylation complicates interpretation of the patterns found in lung tumor and lung cancer cell lines. Unmethylated CpGs which fall in a predicted nucleosome (nuc 3) were, however, observed in the 98 clones from 7 lung cancer cell lines with little or no Cadm1 gene expression. (TIF) [file pone.0038531.s004.tif]

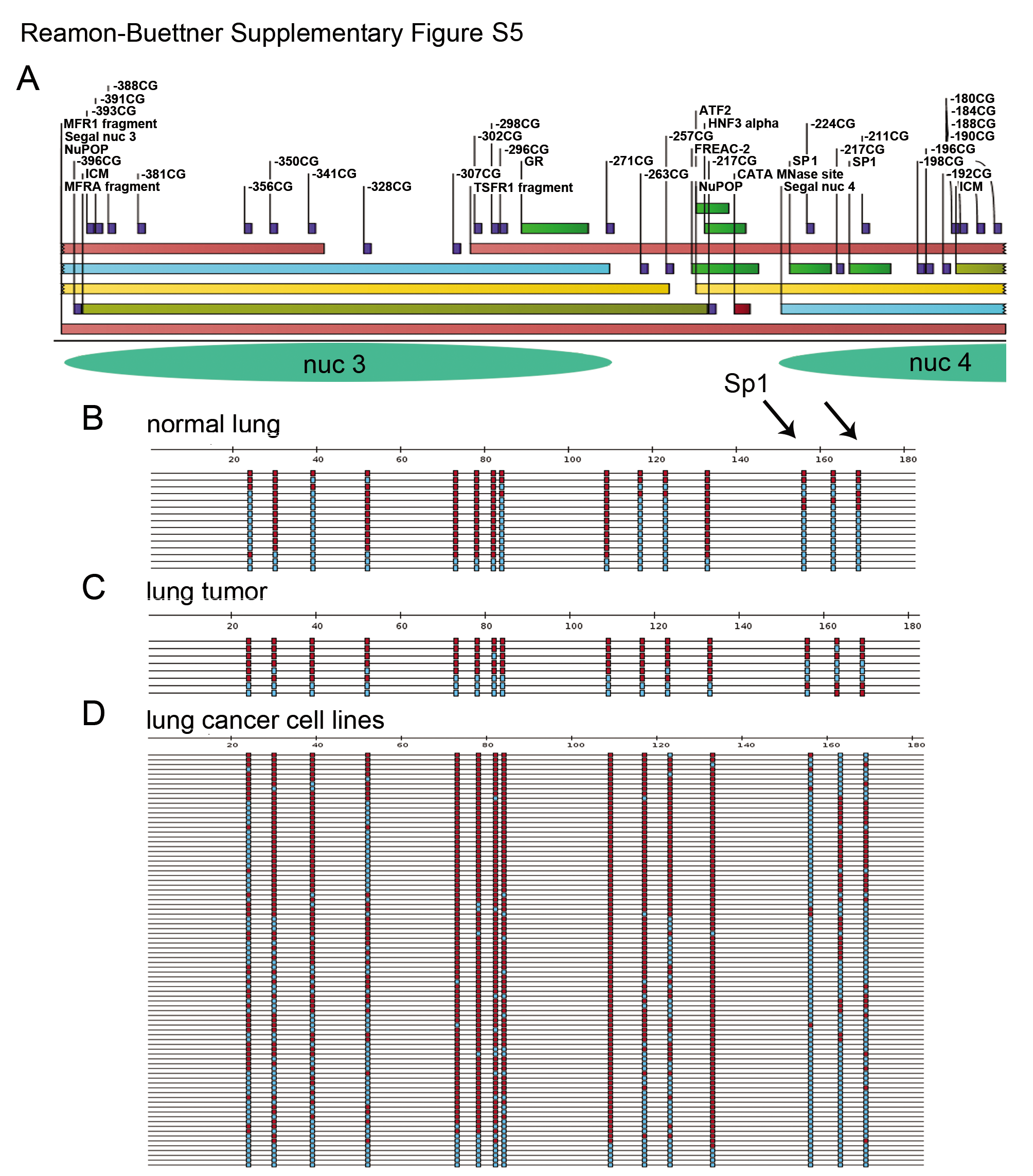

Supplement: Figure S5 — M.Sss I maps in normal lung, lung tumor and lung cancer cell lines in CpGs within the MFRA fragment (222 bp, 27 CpGs, −396 to −180). (A) Annotation of the MFRA fragment showing the CpGs, predicted nucleosomes with the Segal, ICM, NuPOP algorithms, and putative binding sites of lung-specific transcription factors. The maps (B–D) were obtained with BISMA, where blue boxes representing unmethylated CpGs ( = protected) while red boxes, methylated CpGs. In lung tumors and lung cancer cell lines, CpG methylation could be endogenous and/or from the M.SssI treatment. The CpG in the core sequence of two Sp1 sites are indicated by arrows. (B) In normal lung, DNA methylation patterns suggest absence of nucleosome occupancy and possible transcription-factor binding. But clones are also present with a stretch of unmethylated CpGs that are located in a predicted nucleosome (nuc 3). (C–D) Endogenous DNA methylation complicates interpretation of the patterns found in lung tumor and lung cancer cell lines, but clones are present with a stretch of unmethylated CpGs that are located in a predicted nucleosome (nuc 3). In the 86 clones from 7 lung cancer cell lines with little or no Cadm1 gene expression, the CpGs in the Sp1 binding sites at −224 and −211 were mostly unmethylated, which could be both due to Sp1 binding and nucleosome sliding. (TIF) [file pone.0038531.s005.tif]

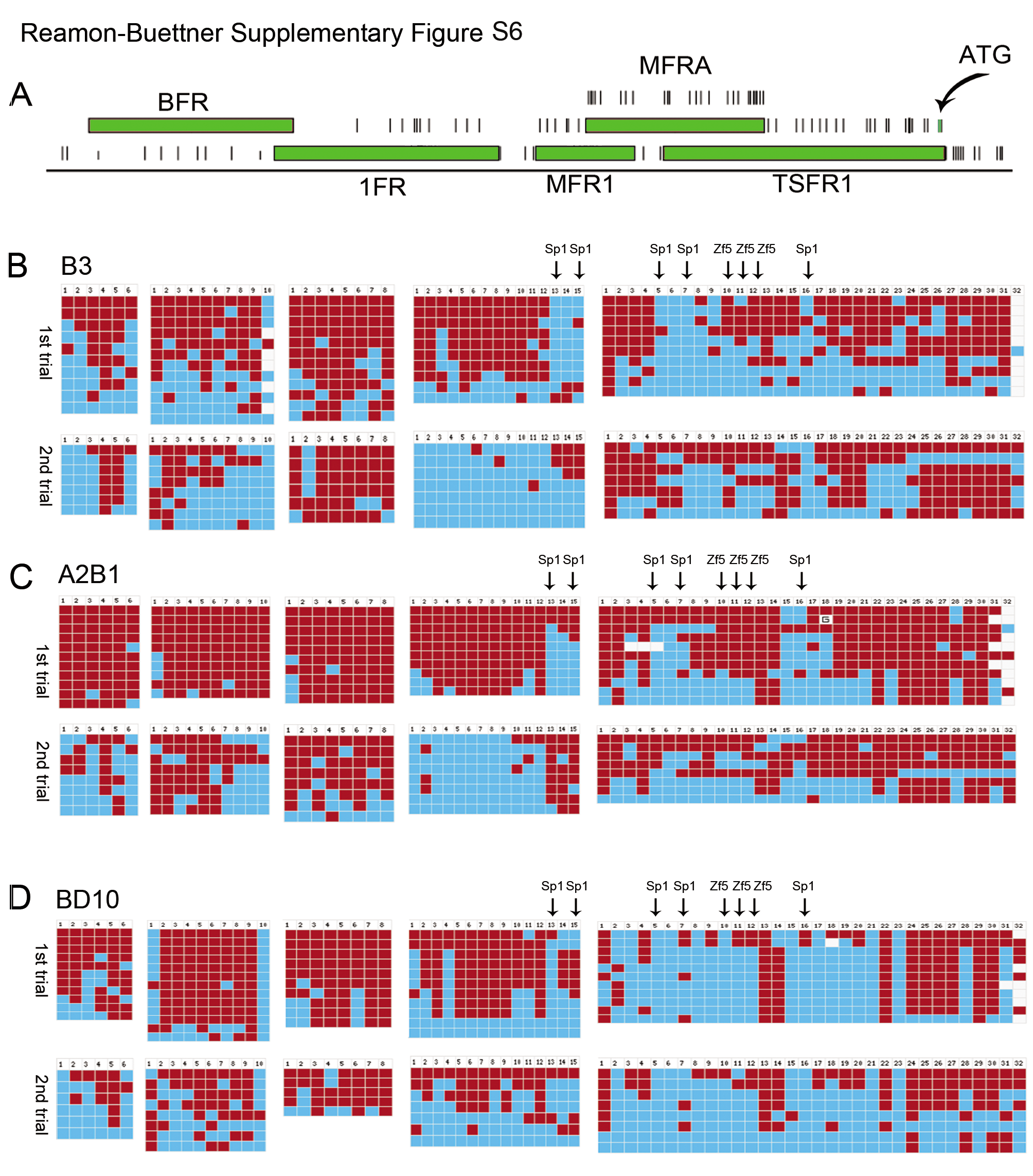

Supplement: Figure S6 — M.Sss I maps in first and second trials in three lung cancer cell lines (B3, A2B1 and BD10). (A) Location of the five fragments analyzed in the Cadm1 promoter region that cover 69 CpGs −944 to +41, relative to the translation start site, ATG. The maps (B–D) were obtained with BISMA, where blue boxes representing unmethylated CpGs ( = protected) while red boxes, methylated CpGs. The fragments are presented with respect to their location i.e. from BFR to TSFR1. In the lung cancer cell lines, CpG methylation could be endogenous and/or from the M.SssI treatment. The lung cancer cell lines (B3, A2B1 and BD10) still express Cadm1, with BD10 the lowest. The CpGs in the core sequence of Sp1 and Zf5 binding sites are indicated by arrows. (TIF) [file pone.0038531.s006.tif]

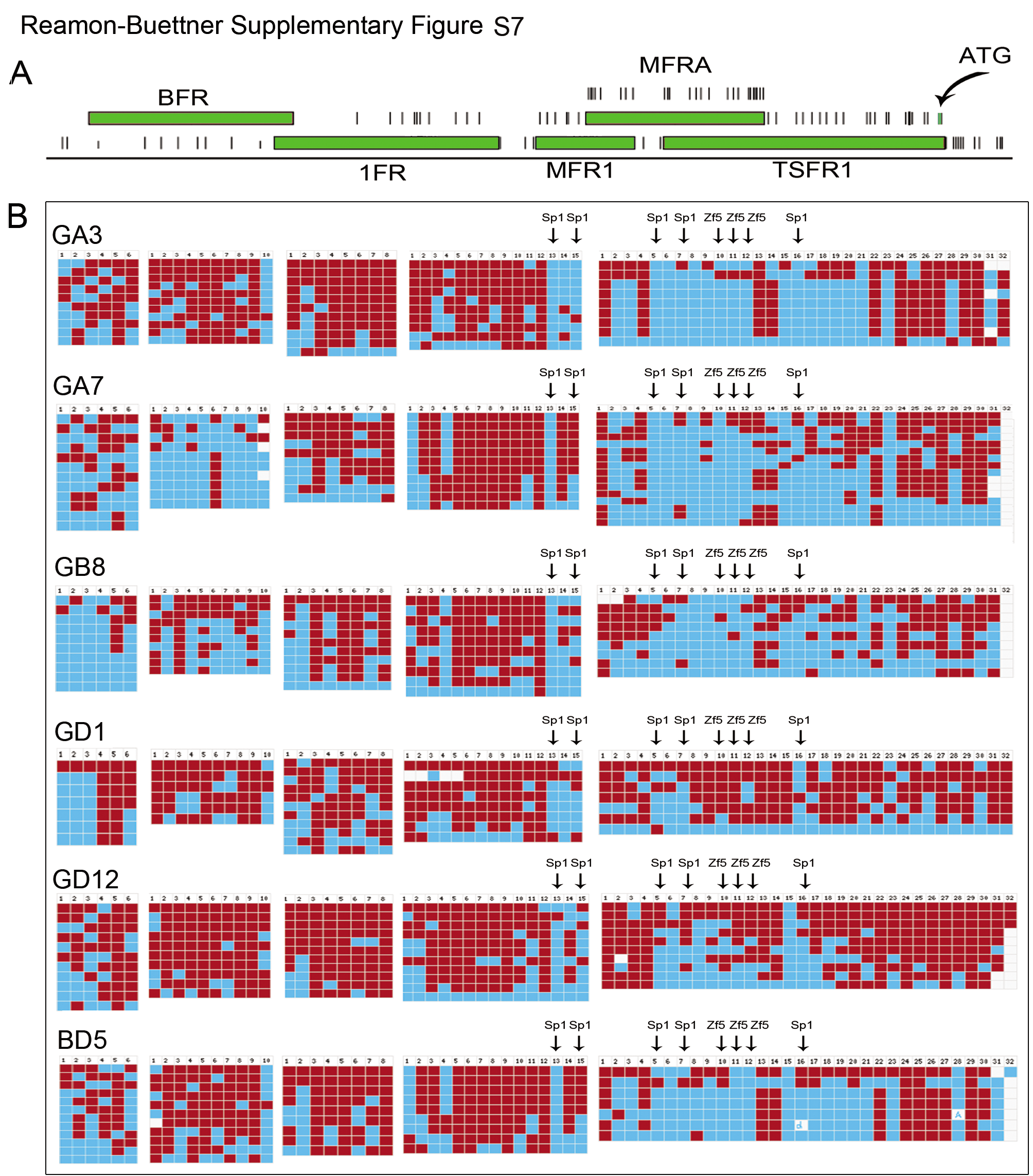

Supplement: Figure S7 — M.Sss I maps in six lung cancer cell lines with little or no Cadm1 gene expression. (A) Location of the five fragments analyzed in the Cadm1 promoter region that cover 69 CpGs −944 to +41, relative to the translation start site, ATG. CpGs are represented by stripes. (B) Methylation maps were obtained with BISMA, where blue boxes representing unmethylated CpGs ( = protected) while red boxes, methylated CpGs. The fragments are presented with respect to their location i.e. from BFR to TSFR1. In the lung cancer cell lines, CpG methylation could be endogenous and/or from the M.SssI treatment. The CpGs in the core sequence of Sp1 and Zf5 binding sites are indicated by arrows. (TIF) [file pone.0038531.s007.tif]

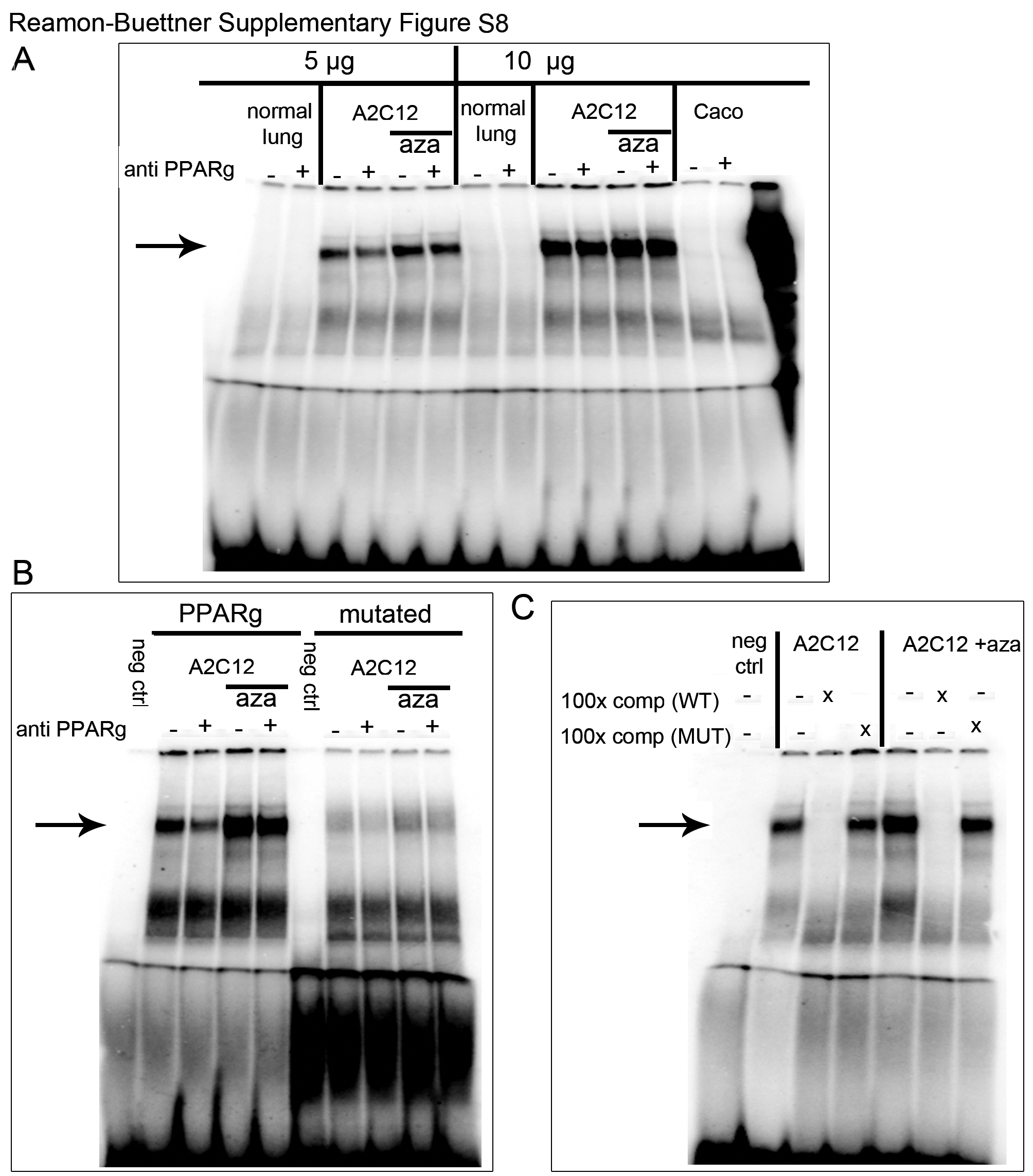

Supplement: Figure S8 — EMSA experiments with PPARg. (A) The predicted PPARg binding sequence in the Cadm1 promoter was used as a probe in nuclear extracts from normal lung, a lung cancer cell line with no Cadm1 gene expression (A2C12), the cell line A2C12 treated with 5-aza-2′-deoxycytidine, and a Caco cell line used as control. No binding was observed in normal lung and in the Caco cell line. In A2C12, where binding occurred, no clear supershift was observed after addition of PPARg antibody, but the band (arrow) became weak as compared to the sample without the antibody. (B) Mutated PPARg core sequence led to abolition of binding. (C) 100x competition with the wild type probe also abolished binding. Negative controls were A2C12 nuclear extracts with no added probes. EMSA probes: WT_F 5′ tctcgcggtcagactctccgacca 3′, WT_R 5′ tggtcggagagtctgaccgcgaga 3′, MUT_F 5′tctcgctggctgactctccgacca 3′, MUT_R 5′ tggtcggagagtcagccagcgaga 3′. Antibody PPARgamma (H-100) sc-7196X Sta Cruz Biotechnology. (TIF) [file pone.0038531.s008.tif]

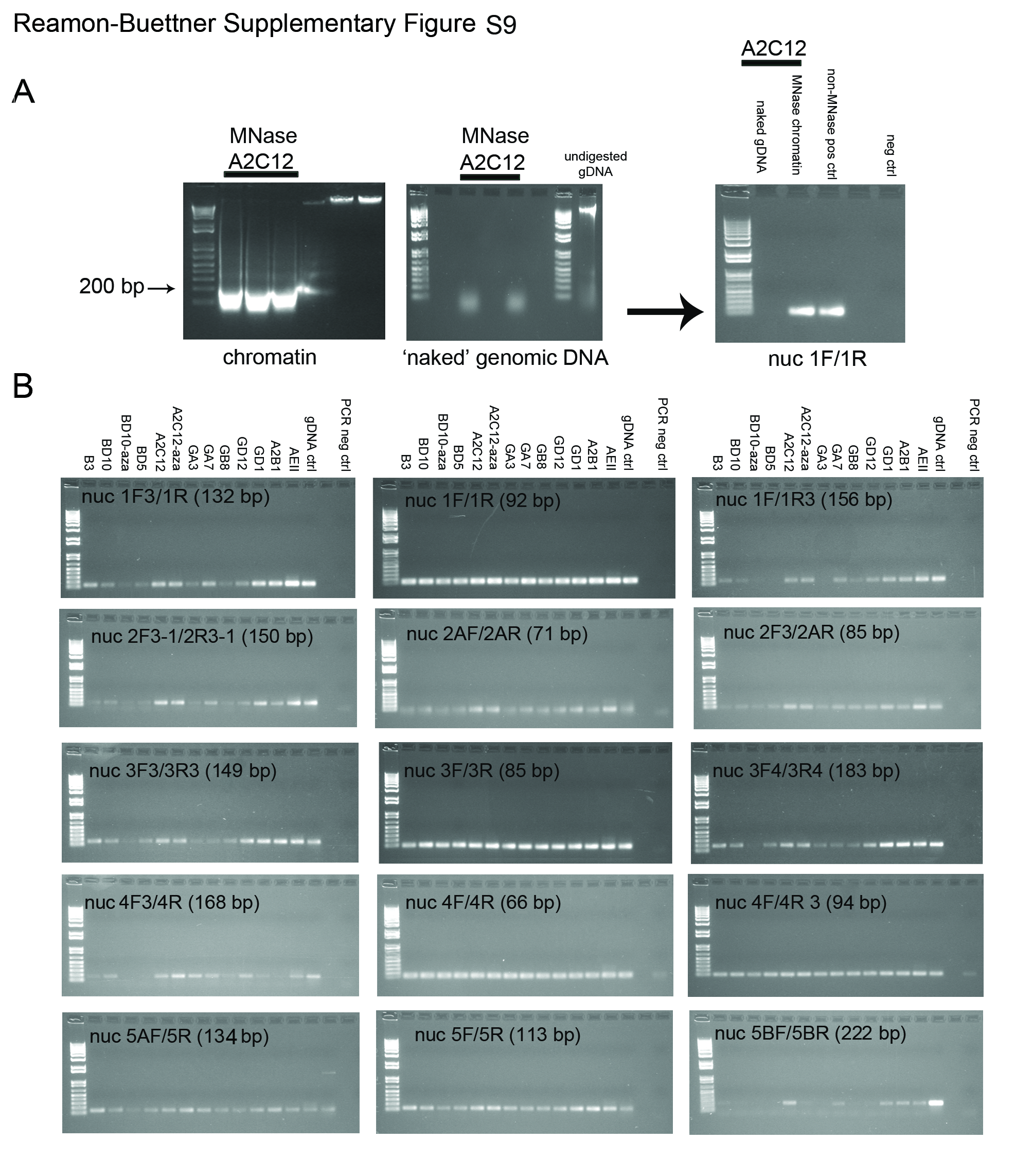

Supplement: Figure S9 — Chromatin analysis with micrococcal nuclease (MNase ) in mouse lung cancer cell lines. (A) DNA fragments after MNase digestion of chromatin and ‘naked’ genomic DNA in a lung cancer cell line (A2C12). No PCR product was obtained in the ‘naked’ genomic DNA (right panel). (B) Normal PCR products with different primers designed on predicted nucleosomes and 2 µL of MNase-digested chromatin as template in the lung cancer cell lines. The samples were analyzed and loaded onto the gel in the same order as given above. Shown are also two cell lines that were treated with 5-aza-dC, and a ‘blind’ control uncharacterized cell line (AEII) which does not express Cadm1. On the upper left corner of each gel are the primer pairs and the size of products. The quantity of PCR products of ‘middle’ primers (middle panel) was higher than those in which one primer is moved towards the left or right border of a nucleosome (left and right panels, respectively). Undigested genomic DNA from a lung cancer cell line (GA3) was used as positive control. (TIF) [file pone.0038531.s009.tif]

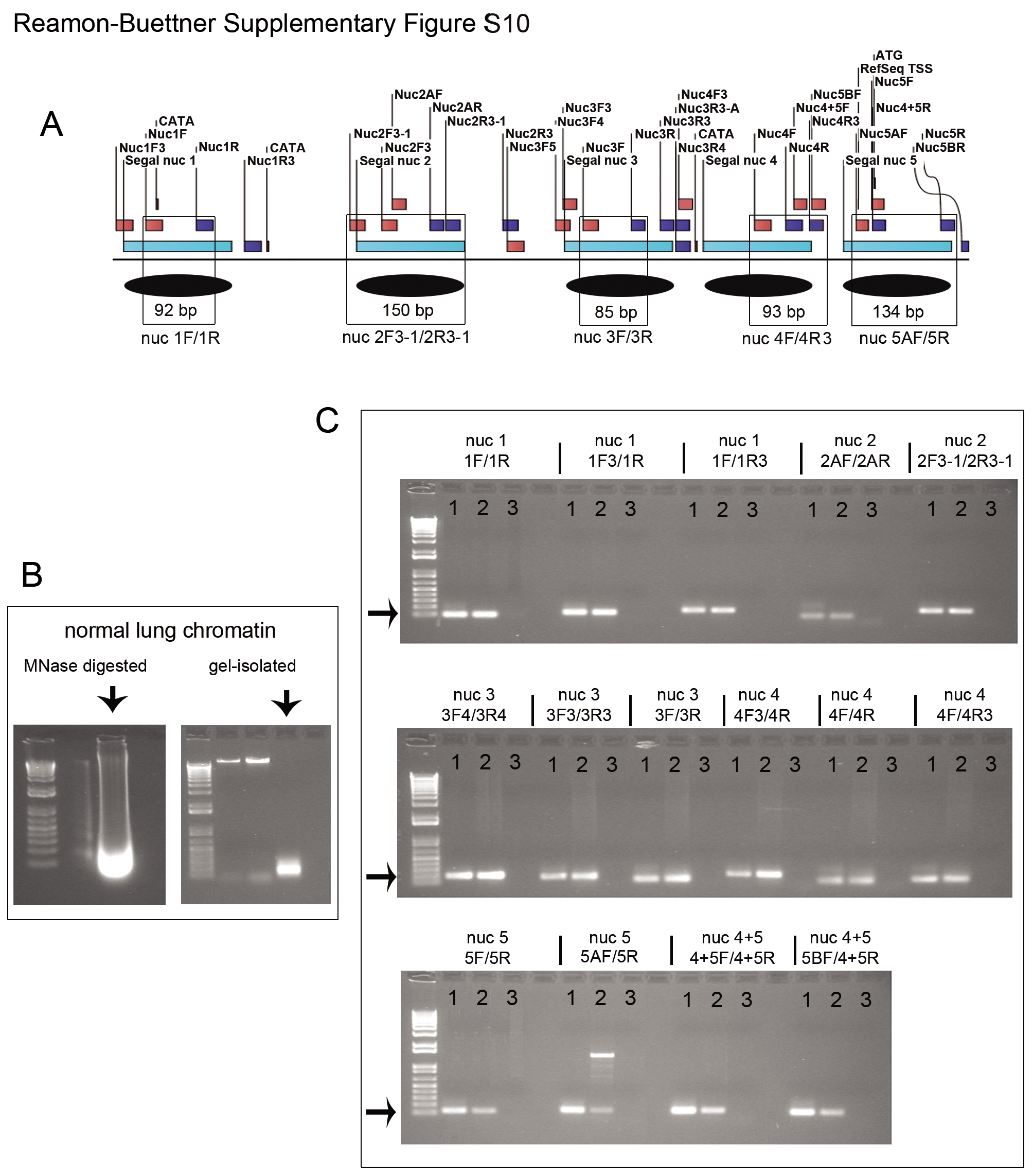

Supplement: Figure S10 — Chromatin analysis with micrococcal nuclease (MNase) in mouse normal lung. (A) Position of five predicted nucleosomes obtained with the Segal lab algorithm, the location of PCR primers used in amplifying fragments after digestion of chromatin with MNase, and MNase-preferred sites (CATA). (B) DNA fragment after MNase digestion of chromatin from seven pooled normal lungs. The quality and concentration of DNA was checked on 1% ethidium bromide gel before performing PCR in (C). Normal PCR products with different primers designed on predicted nucleosomes and 2 µL of MNase-digested chromatin as template. 1: normal lung chromatin, 2: undigested genomic DNA from a lung cancer cell line (A2C12) as positive control, 3: PCR negative control. The primer pair nuc5AF/5R amplified an additional fragment in undigested genomic DNA. (TIF) [file pone.0038531.s010.tif]

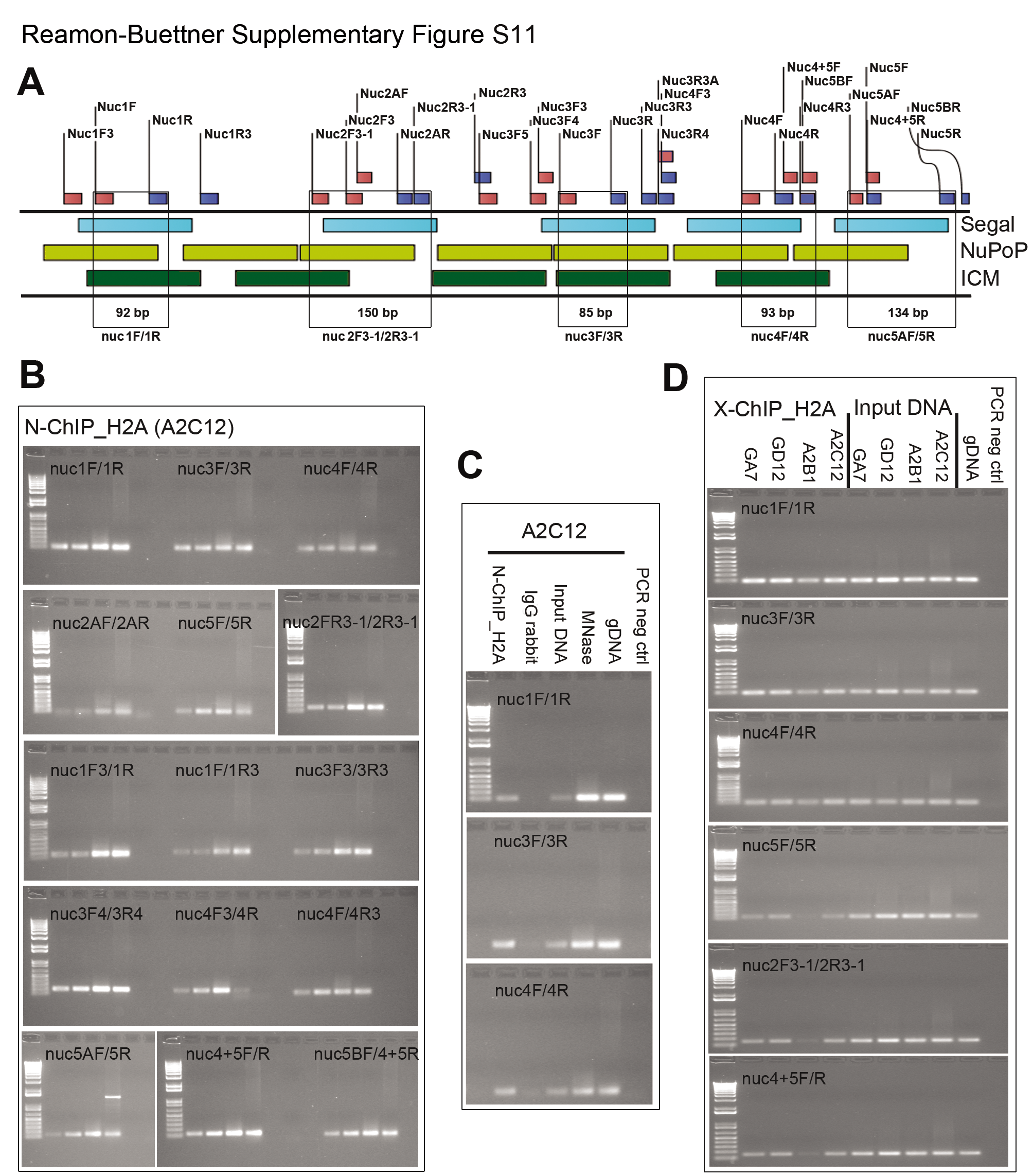

Supplement: Figure S11 — ChIP experiments with H2A using native and crosslinked chromatin in lung cancer cell lines. (A) Position of predicted nucleosomes obtained by different algorithms, location of primers and examples of product size of amplified fragments. (B) Different products from normal PCR and 2 µL of ChIP DNA as template following N-ChIP in A2C12. Loaded onto gel from left in each primer pair after the size marker (Kb ladder), 1–2: ChIP with different chromatin isolation batches, 3: gel-isolated MNase-digested chromatin, 4: undigested genomic DNA control. 5: PCR negative control. The primer pair nuc5AF/5AR amplifies an additional bigger fragment in undigested genomic DNA. (C) An independent N-ChIP experiment with A2C12. (D) X-ChIP with different cell lines showing amplification of same fragments in selected primer pairs. Less PCR product was obtained with A2B1 which still expresses Cadm1, as compared to those without expression. (TIF) [file pone.0038531.s011.tif]

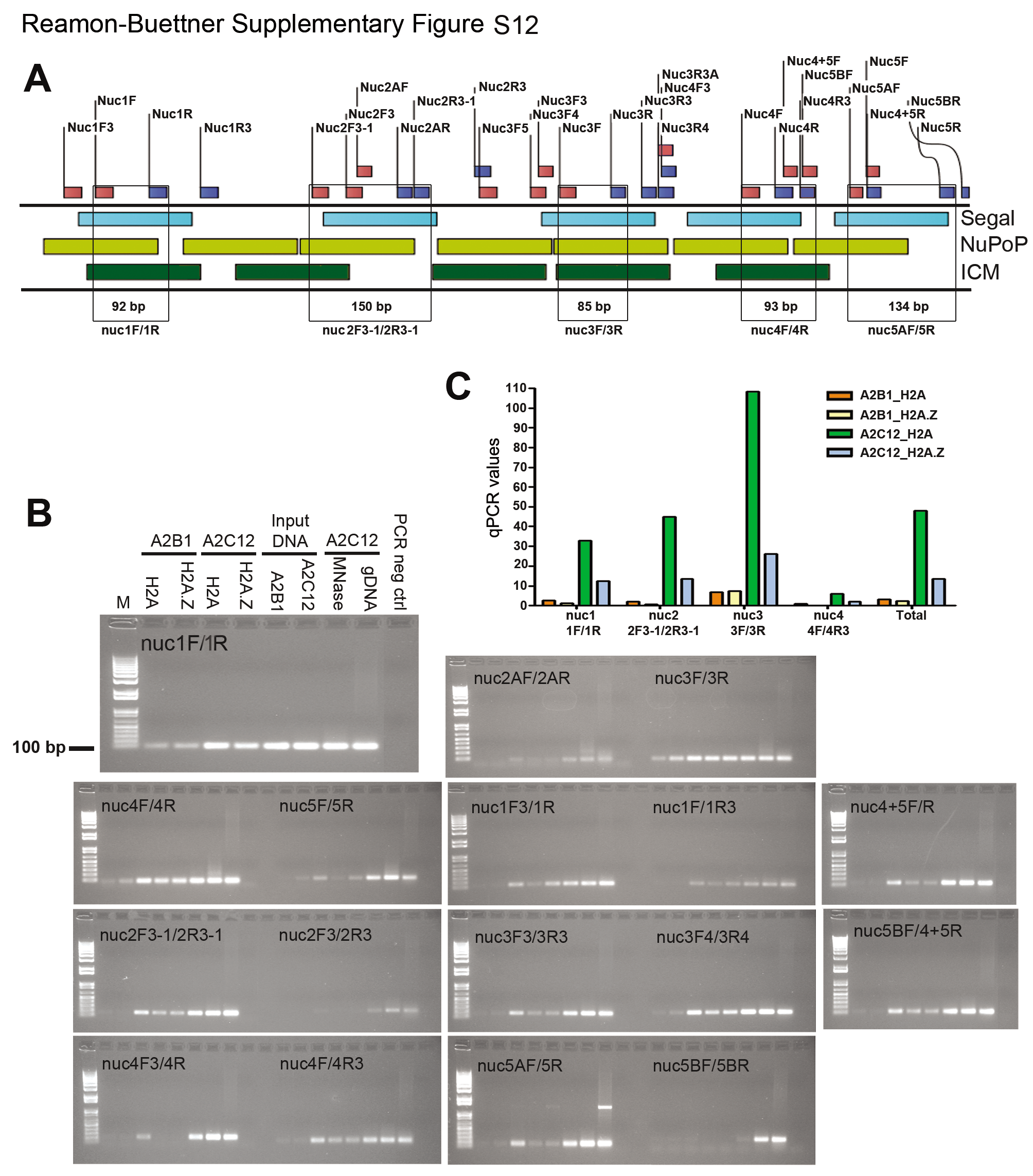

Supplement: Figure S12 — N-ChIP experiments with H2A and H2A.Z in lung cancer cell lines (A2B1 vs. A2C12). (A) Position of predicted nucleosomes obtained by different algorithms, location of primers and examples of product size of amplified fragments. (B) Different products from normal PCR and 2 µl of ChIP DNA as template following ChIP with A2B1 and A2C12. Samples were loaded onto gel as shown for the primer pair nuc1F/1R. The primer pair nuc5AF/5AR amplifies an additional bigger fragment in undigested genomic DNA. Some products are already absent in A2B1. (C) Corresponding qPCR with selected primers, using 20 ng of ChIP DNA as template; results are raw measurements. (TIF) [file pone.0038531.s012.tif]

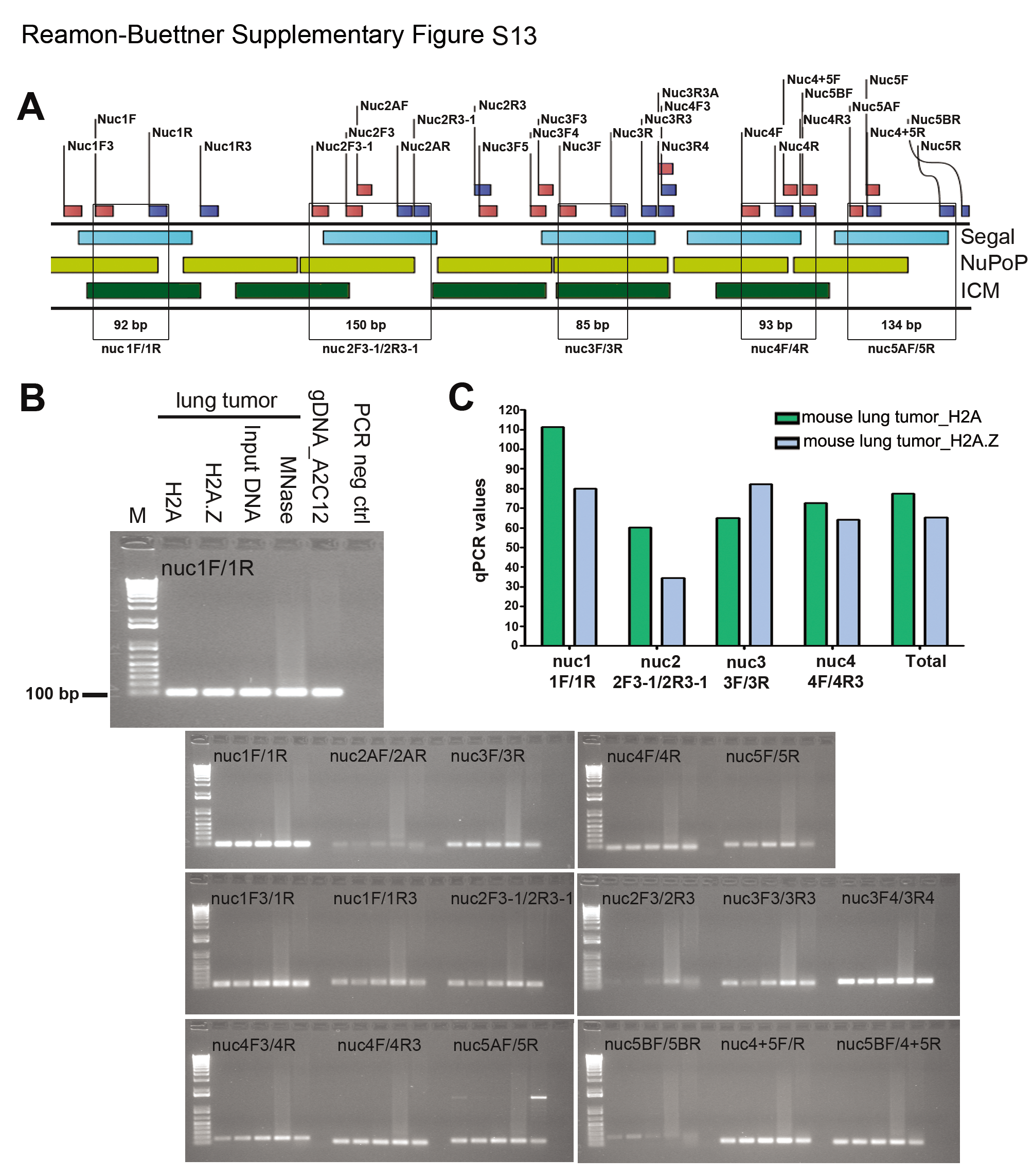

Supplement: Figure S13 — N-ChIP experiments with H2A and H2A.Z in mouse lung tumor. (A) Position of predicted nucleosomes obtained by different algorithms, location of primers and examples of product size of amplified fragments. (B) Different products from normal PCR and 2 µL of ChIP DNA as template following ChIP with lung tumor. Samples were loaded onto gel as shown for the primer pair nuc1F/1R. The primer pair nuc5AF/5AR amplifies an additional bigger fragment in undigested genomic DNA. (C) Corresponding qPCR with selected primers, using 20 ng of ChIP DNA as template. (TIF) [file pone.0038531.s013.tif]

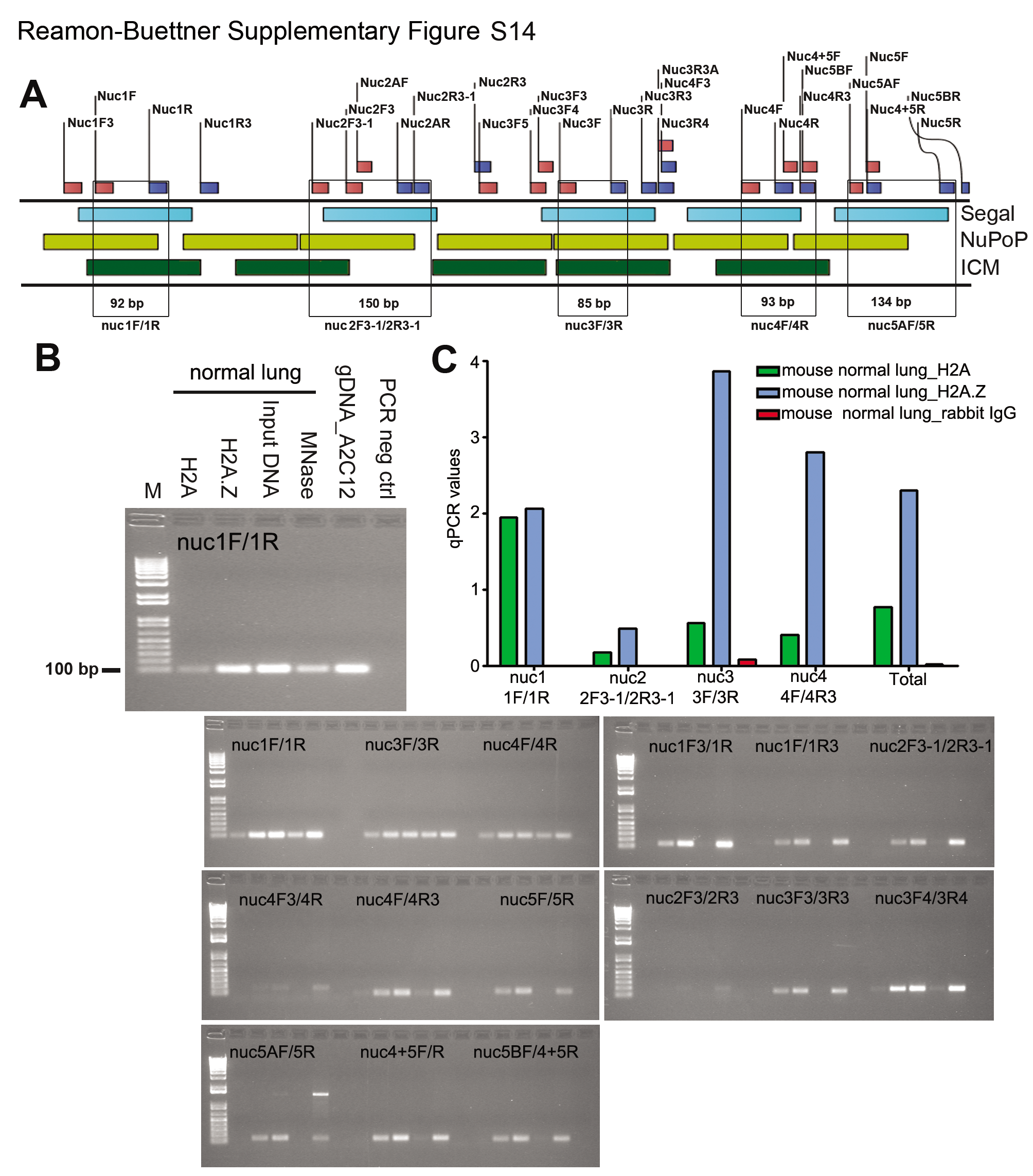

Supplement: Figure S14 — N-ChIP experiments with H2A and H2A.Z in mouse normal lung. (A) Position of predicted nucleosomes obtained by different algorithms, location of primers and examples of product size of amplified fragments. (B) Different products from normal PCR and 2 µL of ChIP DNA as template following ChIP with chromatin isolated from seven pooled normal lungs. In this experiment, the parallel MNase-digested chromatin used as control was not optimal. Nonetheless, the Input DNA is basically the same as the MNase control. Samples were loaded onto gel as shown for the primer pair nuc1F/1R. Some primer pairs gave weak products even in the positive control (undigested genomic DNA A2C12). The primer pair nuc5AF/5AR amplifies an additional bigger fragment in undigested genomic DNA. (C) Quantitative PCR with selected primers, using 20 ng of ChIP DNA as template. The ChIP DNA as measured by the qPCR was obtained from an independent experiment. (TIF) [file pone.0038531.s014.tif]

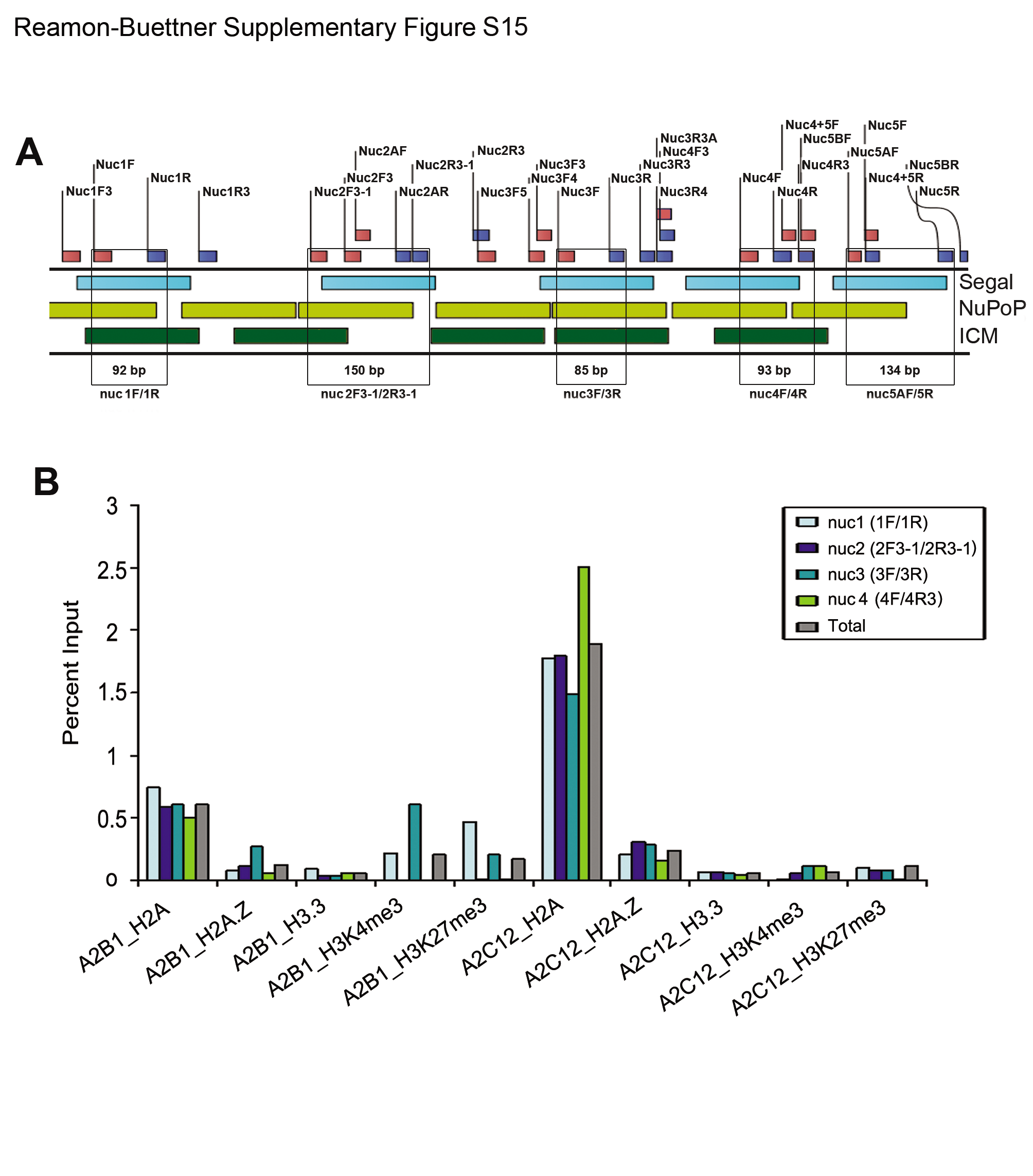

Supplement: Figure S15 — Comparison of chromatin status between A2B1 and A2C12 (A) Position of predicted nucleosomes obtained by different algorithms, location of primers and examples of product size of amplified fragments. (B) Comparison of ChIP DNA with a canonical histone (H2A), histone variants (H3.3, H2A.Z) and histone modifications (H3K4me3, H3K27me3), in different fragments analyzed by qPCR. ChIP results are expressed as Percent Input using Ct values. In A2B1, not all fragments in ChIP with H3K4me3 and H3K27me3 could be amplified. (TIF) [file pone.0038531.s015.tif]

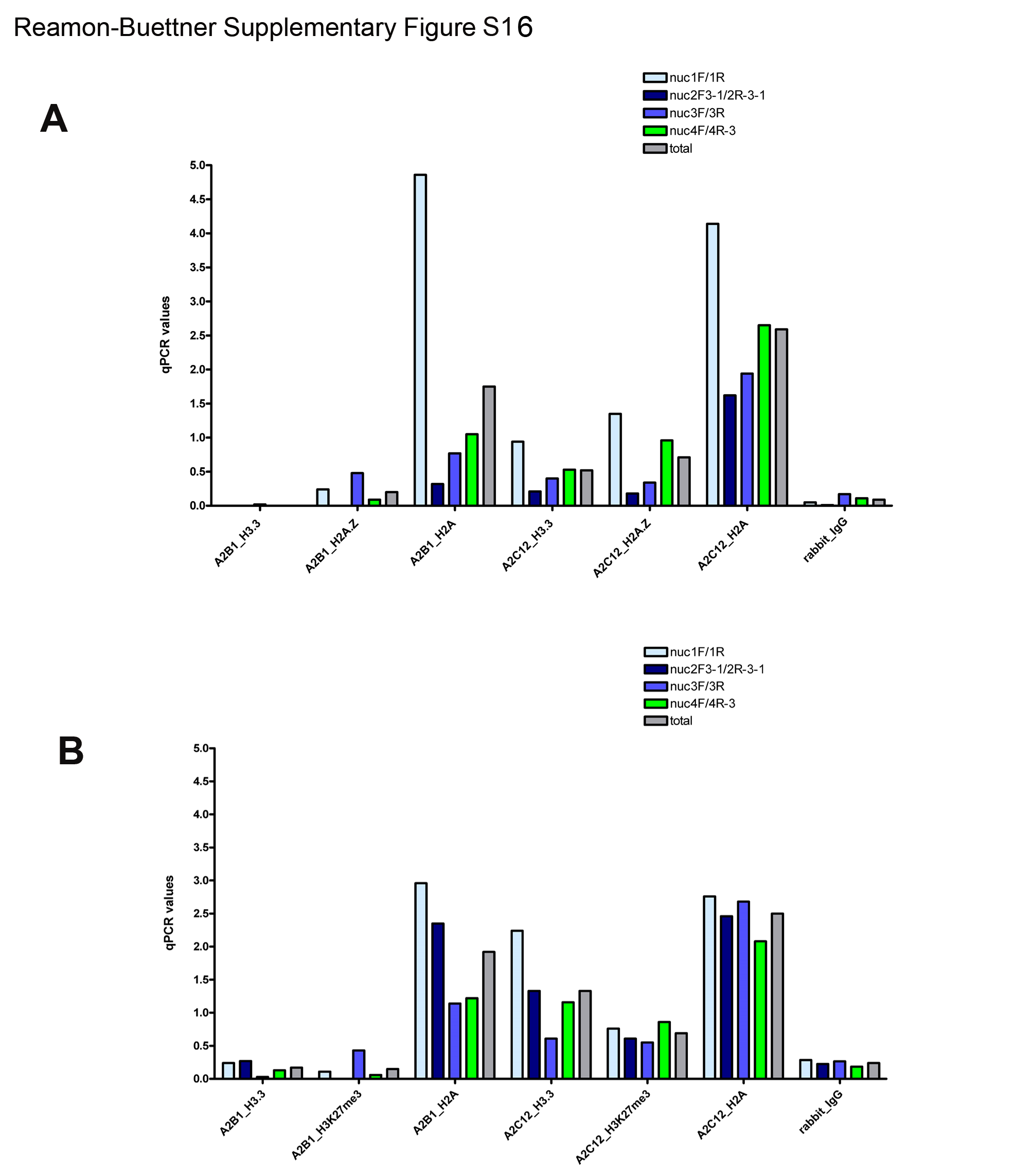

Supplement: Figure S16 — Comparison of different histones between A2B1 and A2C12 (A–B). Examples of ChIP experiments carried out on the same day in A2B1 and A2C12 in different fragments analyzed by qPCR. The result is based on qPCR values obtained with 20 ng of ChIP DNA as template and using calibration standards of a dilution series of gel-isolated MNase-digested chromatin of A2C12. (TIF) [file pone.0038531.s016.tif]
