# Supplementary material for: Dissecting Epigenetic Silencing Complexity in the Mouse Lung Cancer Suppressor Gene Cadm1
Source: PLoS One. 2012 Jun 6;7(6):e38531. doi: 10.1371/journal.pone.0038531 (PMC3368868; doi:10.1371/journal.pone.0038531)
Supplement: Table S1 — Amplified fragments in the Cadm1 promoter region to analyze CpG methylation in bisulfite-treated genomic DNA. (DOC) [file pone.0038531.s017.doc]

**Table S1.** Amplified fragments in the *Cadm1* promoter region to analyze CpG methylation in bisulfite-treated genomic DNA.

| Name of  fragment | Fragment size  (bp) | Location of CpGs (relative to ATG) | No. of CpGs |
| --- | --- | --- | --- |
|  |  |  |  |
| BFR | 255 | -944 to -837 | 6 |
|  |  |  |  |
| 1FR | 279 | -682 to -531 | 10 |
|  |  |  |  |
| MFR1 | 124 | -456 to -341 | 14 |
|  |  |  |  |
| MFRA | 222 | -396 to -180 | 27 |
|  |  |  |  |
| TSFR1 | 345 | -302 +41 | 37 |
|  |  |  |  |
