# Supplementary material for: Dissecting Epigenetic Silencing Complexity in the Mouse Lung Cancer Suppressor Gene Cadm1
Source: PLoS One. 2012 Jun 6;7(6):e38531. doi: 10.1371/journal.pone.0038531 (PMC3368868; doi:10.1371/journal.pone.0038531)
Supplement: Table S2 — Primer sequences used during MNase and ChIP experiments to interrogate nucleosome positioning in the promoter region of mouse Cadm1 gene. (DOC) [file pone.0038531.s018.doc]

**Table S2.** Primer sequences used during MNase and ChIP experiments to interrogate nucleosome positioning in the promoter region of mouse *Cadm1* gene.

| Primer designation | Primer sequence (5’-3’) |
| --- | --- |
| Cadm1-Nuc1F | GAAAGGACTGGAAGCATAGGAGAG |
| Cadm1-Nuc1R | AATGTAGGGAACCGAGGCAGTGAT |
| Cadm1-Nuc1F3 | cacaactaaagtgaggacctgttg |
| Cadm1-Nuc1R3 | ttaagattacacgcacacaaaagc |
| Cadm1-Nuc2AF | tagtctgccgctaggctgtt |
| Cadm1-Nuc2AR | agagaagtgcatgcgtgctt |
| Cadm1-Nuc2F3 | ttccaactatcccctagtctgc |
| Cadm1-Nuc2R3 | aaaatttcctttccctcacctt |
| Cadm1-Nuc2F3-1 | atccacttgaaaatcccagaac |
| Cadm1-Nuc2R3-1 | cccggattgacaaaacaatct |
| Cadm1-Nuc3F | GCGGCGAGTGGCGTAAAGGTTC |
| Cadm1-Nuc3R | GTGGCTGGGGAGGGGGTTGC |
| Cadm1-Nuc3F3 | gtcagactctccgaccagga |
| Cadm1-Nuc3R3 | ccgaaggaggagaacctgta |
| Cadm1-Nuc3F4 | gaatctcgcggtcagactctc |
| Cadm1-Nuc3R4 | gttaaaattcggcctcgcttc |
| Cadm1-Nuc4F | GGTGGGGGAGGGGGCTAGTTCTCG |
| Cadm1-Nuc4R | AGGAGCGACCAATCGTGGCACCTC |
| Cadm1-Nuc4F3 | cgaggccgaattttaacgta |
| Cadm1-Nuc4R3 | gctaatgagatgcgctggag |
| Cadm1-Nuc5F | GACATGGCGAGTGCTGTG |
| Cadm1-Nuc5R | GAAAGGAGCAACAGCAGGAG |
| Cadm1-Nuc5AF | gccaGTCTGAGGCAGGTG |
| Cadm1-Nuc5BF | CAGCGcatctcattagcatc |
| Cadm1-Nuc5BR | cctacctgtgggGATCAGTG |
| Cadm1-Nuc4+5F | ATTGGTCGCTCCTGACTCC |
| Cadm1-Nuc4+5R | AGCACAGCACTCGCCATGT |
