# Supplementary material for: Dissecting Epigenetic Silencing Complexity in the Mouse Lung Cancer Suppressor Gene Cadm1
Source: PLoS One. 2012 Jun 6;7(6):e38531. doi: 10.1371/journal.pone.0038531 (PMC3368868; doi:10.1371/journal.pone.0038531)
Supplement: Table S3 — Primer combinations and products used during MNase and ChIP experiments to interrogate nucleosome positioning in the promoter region of mouse Cadm1 gene. (DOC) [file pone.0038531.s019.doc]

**Table S3.** Primer combinations and products used during MNase and ChIP experiments to interrogate nucleosome positioning in the promoter region of mouse *Cadm1* gene. Listed are only primer combinations that gave PCR products in both chromatin and genomic DNA and used throughout the study.

| Predicted nucleosome | Primer  Combination | Product  size (bp) | Arbitrary  designation  nucleosome | Location  *Cadm1* promoter,  relative to ATG |
| --- | --- | --- | --- | --- |
| Nuc 1 | Cadm1-Nuc1F/Cadm1-Nuc1R | 92 | middle | -981 to -890 |
|  | Cadm1-Nuc1F3/ Cadm1-Nuc1R | 132 | left | -1021 to -890 |
|  | Cadm1-Nuc1F/ Cadm1-Nuc1R3 | 156 | right | -981 to -825 |
| Nuc 2 | Cadm1-Nuc2AF/ Cadm1-Nuc2AR | 71 | middle | -649 to -579 |
|  | Cadm1-Nuc2F3-1/ Cadm1-Nuc2R3-1 | 150 | left | -706 to -557 |
|  | Cadm1-Nuc2F3/ Cadm1-Nuc2AR | 85 | right | -663 to -579 |
| Nuc 3 | Cadm1-Nuc3F/ Cadm1-Nuc3R | 85 | middle | -392 to -308 |
|  | Cadm1-Nuc3F3/ Cadm1-Nuc3R3 | 149 | left | -417 to -269 |
|  | Cadm1-Nuc3F4/ Cadm1-Nuc3R4 | 183 | right | -429 to -247 |
| Nuc 4 | Cadm1-Nuc4F/ Cadm1-Nuc4R | 66 | middle | -161 to -96 |
|  | Cadm1-Nuc4F3/ Cadm1-Nuc4R | 168 | left | -263 to -96 |
|  | Cadm1-Nuc4F/ Cadm1-Nuc4R3 | 94 | right | -263 to -68 |
| Nuc 5 | Cadm1-Nuc5F/ Cadm1-Nuc5R | 113 | middle | -3 to +110 |
|  | Cadm1-Nuc5AF/ Cadm1-Nuc5R | 134 | left | -24 to + 110 |
|  | Cadm1-Nuc5BF/ Cadm1-Nuc5BR | 222 | left/right | -84 to + 138 |
| Nuc 4+5 | Cadm1-Nuc4+5F/ Cadm1-Nuc4+5R | 125 | in between | -108 to +17 |
|  | Cadm1-Nuc5BF/ Cadm1-Nuc4+5R | 101 | in between | -84 to +17 |
