# Supplementary material for: Dissecting Epigenetic Silencing Complexity in the Mouse Lung Cancer Suppressor Gene Cadm1
Source: PLoS One. 2012 Jun 6;7(6):e38531. doi: 10.1371/journal.pone.0038531 (PMC3368868; doi:10.1371/journal.pone.0038531)
Supplement: Methods S1 — Chromatin immunoprecipitation (ChIP): X-ChIP. (DOC) [file pone.0038531.s020.doc]

# Methods S1

## Chromatin immunoprecipitation (ChIP): X-ChIP

Formaldehyde was added directly to the cell culture media at a final concentration of 1 %. Fixation was undertaken at room temperature for 10 min and stopped by adding glycine to a final concentration of 0.125 M. After crosslinking, cells were collected by centrifugation and rinsed with cold PBS. Cell pellets were resuspended in lysis buffer (5 mM PIPES pH 8.0, 85 mM KCl, 0.5% NP40, 1mM DTT, 1 mg/mL pefabloc, 10 µg/mL leupeptin, and 10 µl/mL aprotinin) and incubated on ice for 20 min. Nuclei were collected by microcentrifugation and resuspended in sonication buffer (50 mM Tris-HCl pH 8.1, 10 mM EDTA, 1% SDS, 1mM DTT, 1 mg/mL pefabloc, 10 µg/mL leupeptin, and 10 µl/mL aprotinin) and incubated on ice for 10 min. Prior to sonication, 0.1 g glass beads (212- 300 µm diameter, Sigma) was added to each sample. The samples were then fragmented on ice using a sonicator (Branson Sonifier W-250 Classic) at setting "output control 4" and "cycle 50%" for five 20 s pulses, to give fragments of about 0.2-1.6 kb, and then microcentrifuged. Protein A-Sepharose CLB4 (Pharmacia, Amersham Biosciences) was blocked with 1 mg/mL BSA and washed extensively before use. Chromatin preparations were precleared by incubation with ‘blocked’ Protein A-Sepharose for 1 h at 4°C. Precleared chromatin from 2.5 x 10^7^ cells was incubated with 5 µg histone antibody or no antibody and rotated at 4°C overnight. After recovering immunocomplexes, extensive washing: 2x with dialysis buffer (2 mM EDTA, 50 mM Tris-HCl pH 8.0, 0.2 % sarkosyl and protease inhibitors) and 4x with IP-wash buffer (100 mM Tris-HCl pH 9.0 , 500 mM LiCl, 1% NP40, 1% deoxycholic acid, and protease inhibitors) and elution (50 mM NaHCO_3_, 1% SDS, shaking on ice for 60 min). Crosslinking was reversed by adding NaCl to a final concentration of 200 mM, and RNA was removed by the addition of 10 µg RNase A per sample followed by incubation at 65°C for at least 5 h. The samples were then precipitated at -20°C overnight by the addition of 2.5 volumes of ethanol and then pelleted by microcentrifugation. The samples were resuspended in 100 µL Tris-EDTA pH 7.5, 25 µL of 5x proteinase K buffer (50 mM Tris-HCl pH 7.5, 25 mM EDTA, 1.25 % SDS) and 1.5 µL of proteinase K (20 µg/ µL), and incubated at 55°C for 2 h. Samples were extracted with phenol-chloroform-isoamyl alcohol (25:24:1) followed by extraction with chloroform-isoamyl alcohol and then precipitated with 1/10 volume of 5 M NaCl, 5 µg glycogen, and 2.5 volumes of ethanol. The pellets were collected by microcentrifugation and resuspended in 30 µL H_2_O.
